# Aromatic Acids and Leucine Derivatives Produced from the Deep-Sea Actinomycetes *Streptomyces* *chumphonensis* SCSIO15079 with Antihyperlipidemic Activities

**DOI:** 10.3390/md20040259

**Published:** 2022-04-07

**Authors:** Ziqi Su, Kunlong Li, Xiaowei Luo, Yongyan Zhu, Shao-Yu Mai, Quanhong Zhu, Bin Yang, Xuefeng Zhou, Huaming Tao

**Affiliations:** 1 Guangdong Provincial Key Laboratory of Chinese Medicine Pharmaceutics, School of Traditional Chinese Medicine, Southern Medical University, Guangzhou 510515, China; ziqisl@163.com (Z.S.); yongyanzhu0521@163.com (Y.Z.); shaoyumai@smu.edu.cn (S.-Y.M.); zqh@smu.edu.cn (Q.Z.); 2 CAS Key Laboratory of Tropical Marine Bio-Resources and Ecology, Guangdong Key Laboratory of Marine Materia Medica, South China Sea Institute of Oceanology, Chinese Academy of Sciences, Guangzhou 510301, China; likunlong16@mails.ucas.ac.cn (K.L.); yangbin@scsio.ac.cn (B.Y.); 3 Southern Marine Science and Engineering Guangdong Laboratory (Guangzhou), Guangzhou 511458, China; 4 Institute of Marine Drugs, Guangxi University of Chinese Medicine, Nanning 530200, China; luoxiaowei1991@126.com

**Keywords:** deep-sea actinomycetes, aromatic acids, oximes, antihyperlipidemic

## Abstract

Six new aromatic acids (**1**–**6**) and three new leucine derivatives containing an unusual oxime moiety (**7**–**9**) were isolated and identified from the deep-sea-derived actinomycetes strain *Streptomyces chumphonensis* SCSIO15079, together with two known compounds (**10**–**11**). The structures of **1**–**9** including absolute configurations were determined by detailed NMR, MS, and experimental and calculated electronic circular dichroism spectroscopic analyses. Compounds **1**–**9** were evaluated for their antimicrobial and cytotoxicity activities, as well as their effects on intracellular lipid accumulation in HepG2 cells. Compounds **3** and **4**, with the most potent inhibitory activity on intracellular lipid accumulation at 10 μM, were revealed with potential antihyperlipidemic effects, although the mechanism needs to be further studied.

## 1. Introduction

Hyperlipidemia, defined as an increase in the blood lipids levels, is a risk factor for cardiovascular diseases, including coronary atherosclerosis, cardiovascular diseases, Heart failure, and other metabolic diseases. Hyperlipidemia is also known as an aggravation of several pathological conditions such as hypothyroidism and chronic renal failure. Globally, approximately 12 million people die each year due to hyperlipidemia [1,2,3,4]. At present, drugs used to reduce the level of blood lipids mainly include statins (atorvastatin, lovastatin, etc.), fibrates (bezafibrate, lifibrate, etc.), and resin (cholestyramine, colestipol, etc.). Nevertheless, these synthetic medicines are often associated with some serious side effects such as diarrhea, nausea, gallstones, myositis, and abnormal liver function [5,6,7]. Therefore, it is urgent to discover new lipid-lowering agents with more therapeutic value and less tolerable side effects from natural products.

For the past several decades, many marine-derived active ingredients have been tested and proven with lipid-lowering or antihyperlipidemic effects, such as the seaweed polysaccharide [8], marine algal polyphenols [9], polyunsaturated fatty acids from oily fish [10,11], and fish protein [12]. Marine-derived microorganisms have shown promising potential to produce a great number of active substances including antibacterial, antiviral, antioxidant, as well as cytotoxic compounds [13,14,15,16,17,18]. Marine extremophilic microorganisms tend to produce fascinating novel types of bioactive secondary metabolites; therefore deep-sea-derived microorganisms seem to be significant sources for discovering lead compounds in drug discovery [19,20]. 

**Figure 1:**
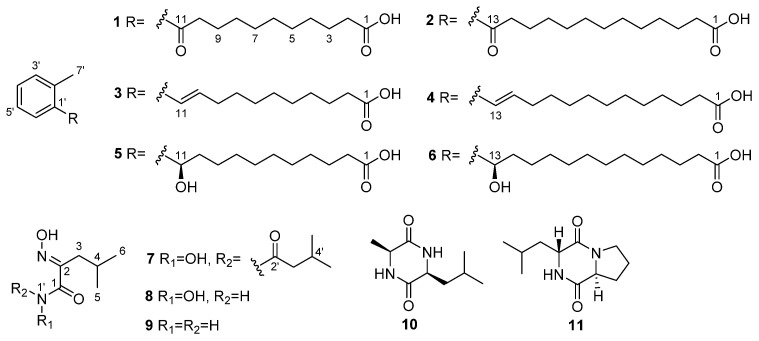
Structures of compounds **1**–**11**.

In our search, six new aromatic acids (**1**–**6**), three new leucine derivatives containing an unusual oxime moiety (**7**–**9**), and two known compounds (**10**, **11**) were obtained from the actinomycetes strain *Streptomyces chumphonensis* SCSIO15079, isolated from a sediment sample of Indian Ocean, with a depth of 3386 m (Figure 1). In order to study the potential antihyperlipidemic effects of these compounds, their effects on intracellular lipid accumulation in HepG2 cells were evaluated. Herein, we describe the fermentation, isolation, structural determination, and biological activity of these compounds. 

## 2. Results and Discussion

### 2.1. Structure Elucidation

**Figure 2:**
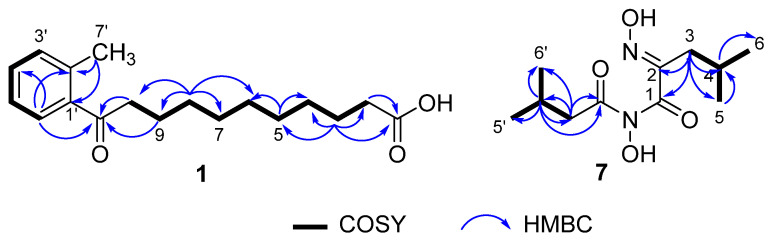
Key COSY and HMBC correlations in **1** and **7**.

Compound **1** was isolated as a yellowish solid. Its molecular formula of C_18_H_26_O_3_ was determined by HRESIMS (*m/z* 289.1809 [M − H]^−^, calcd for, 289.3950), which required 6 degrees of unsaturation. The ^1^H NMR spectrum displayed an ABCD aromatic spin system resonated at *δ*_H_ 7.67 (1H, d, *J* = 7.6 Hz, H-6′), 7.38 (1H, m, H-3′), 7.27 (2H, m, H-4′,5′) for an aromatic ring with vicinal substitution. The ^1^H−^1^H COSY correlations established that C-2 to C-10 were a long-chain alkanes system (Figure 2). The ^13^C NMR spectrum, in combination with DEPT and HSQC spectra, revealed the presence of 18 carbons, including a methyl (C-7′), six aromatic carbons (C-1′ to C-6′), nine methylenes (C-2 to C-10), and two carbonyl carbons (C-1 and C-11). The HMBC correlations from H-5′ (*δ*_H_ 7.27) and H-6′ (*δ*_H_ 7.67) to C-11 (*δ*_C_ 207.4) and C-9 (*δ*_C_ 26.2), respectively, established the connection of the conjugated chain to an aromatic ring. The position of the methyl group in the aromatic ring was determined by HMBC correlations observed from H-7′ (*δ*_H_ 2.42) to C-2′ (*δ*_C_ 138.6) and C-1′ (*δ*_C_ 139.7), which indicated a methylbenzene moiety. As only H-6 showed an HMBC correlation with the ketone signal, it was deduced that the ketone C-11 (*δ*_C_ 207.4, s) must be attached directly to the benzene ring (Figure 2). Thus, the structure of **1** was identified, as shown in Figure 1, and named 11-oxo-11-(o-tolyl) undecanoic acid (**1**). 

Compound **2** was isolated as a yellowish solid. Its molecular formula of C_20_H_30_O_3_ was determined by the HRESIMS (*m/z* 319.2268 [M − H]^−^, calcd for, 319.2273), which required 6 degrees of unsaturation. Its ^1^H and ^13^C NMR data (Table 1 and Table 2) revealed that the aromatic ring with vicinal substitution of **2** was the same as **1**. Compared to **1**, the only difference of **2** was two more methylene groups in the fatty acid chains, the ^1^H NMR data of which showed 11 methylene groups at *δ*_H_ 1.25~1.36. Thus, the structure of **2** was identified as shown in Figure 1 and named 13-oxo-13-(o-tolyl) tridecanoic acid (**2**).

Compound **3** was isolated as a yellowish solid. Its molecular formula of C_18_H_26_O_2_ was determined by the HRESIMS (*m/z* 297.1825 [M + Na]^+^, calcd for, 297.1830), which required 6 degrees of unsaturation. It was determined as a 10,11-unsaturated analogue of **1** based on the presence of the ^1^H-^1^H COSY spectrum correlated two olefinic protons at *δ*_H_ 6.06 (1H, dt, *J* = 15.6, 7.0 Hz, H-10) and 6.57 (1H, d, *J* = 15.6 Hz, H-11), indicating an *E*-disubstituted double bond, which replaced the ketone signal at C-11 of **1**, as evident from the 2D NMR data in association with the HRESIMS data. Thus, the structure of **3** was identified (*E*)-11-(o-tolyl) undec-10-enoic acid (**3**).

Compound **4** was determined to be C_20_H_30_O_2_ by the HRESIMS (*m/z* 325.2138 [M + Na]^+^) data, requiring 6 degrees of unsaturation. A comparison of the NMR data between **4** and **3** (Table 1 and Table 2) revealed that the side chain of **4** possessed two methylene groups, whereas the remaining NMR data of both compounds were closely similar. Thus, the structure of **4** was identified as (*E*)-13-(o-tolyl) undec-12-enoic acid (**4**).

**Figure 3:**
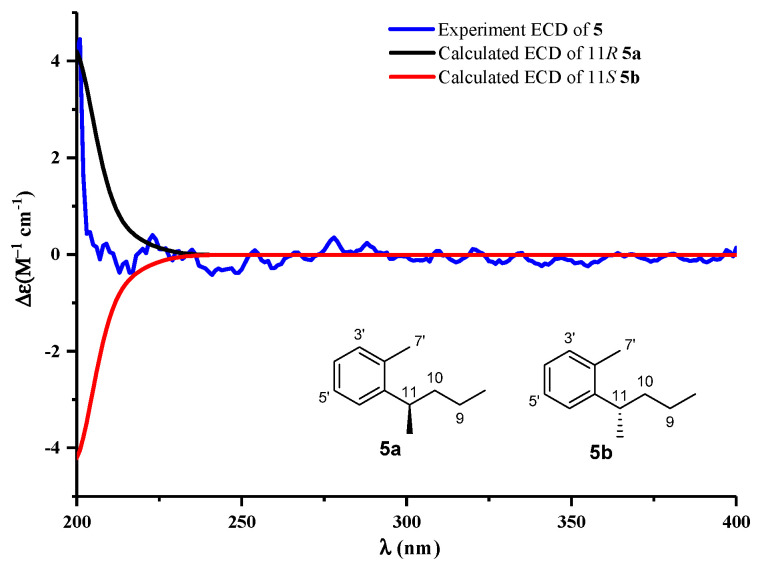
Experimental ECD spectra of **5**, and the calculated ECD spectra of truncated models **5a**/**5b**.

Compound **5** was isolated as a yellowish solid. Its molecular formula of C_18_H_28_O_3_ was determined by the HRESIMS (*m/z* 291.1969 [M − H]^−^, calcd for, 291.1960), which required 5 degrees of unsaturation. ^1^H and ^13^C NMR data (Table 1 and Table 2) of **5** closely resembled those of **1**. The only difference between them was that the ketone at the 11-position in **1** was replaced by an oxygenated methine group (*δ*_H_ 4.86/*δ*_C_ 71.1) in **5**, which was corroborated by the HMBC correlation from H-11 to C-1′ (*δ*_C_ 144.6), 2′ (*δ*_C_ 135.5), and 6′ (*δ*_C_ 126.4). The absolute configuration of **5** was established based on a comparison of its experimental electronic of the 11*R*-**5a** model and the 11*S*-**5b** model at the B3LYP/6-31G (d,p) level in Gaussian 03, and the former was showed relatively good agreement with the experimental one (Figure 3). Thus, the absolute structure of **5** was defined as 11*R*. We also tried employing Mosher’s method to determine the absolute configuration of C-11 in **5**. The treatment of **5** with (*R*)- and (*S*)-MTPA-Cl yielded (*S*)- and (*R*)-MTPA ester derivatives, respectively. However, the values in the ^1^H-NMR spectrum of the mono-(*S*)- and -(*R*)-MTPA esters in **5** had no significant differences. This might be a result from the aliphatic chain being too long to flip. Thus, the structure of **5** was identified to be (*R*)-11-hydroxy-11-(o-tolyl) undecanoic acid (**5**).

Compound **6** showed the molecular formula of C_20_H_32_O_3_ by HRESIMS (*m/z* 319.2279 [M − H]^−^) data, which pointed to an analogue of **5**. The ^1^H and ^13^C NMR spectrum was indeed similar to that of **5** (Table 1 and Table 2), and as expected, the ^13^C NMR data indicated clearly two additional methylene groups (Table 2). Compound **6** was indicated to be *R*-configuration at C-13, and its optical rotation was ([α]${}_{D}^{25}$ +2.6 (c 0.10, MeOH)), compared with compound **5** ([α]${}_{D}^{25}$ +4.0 (c 0.10, MeOH)). Thus, the structure of **6** was identified to be (*R*)-11-hydroxy-11-(o-tolyl) undecanoic acid (**6**). 

Compound **7** was afforded as a yellowish powder. Its molecular formula of C_11_H_20_N_2_O_4_ was inferred from HRESIMS (*m/z* 245.1496 [M + H]^+^, calcd for, 245.2990) spectrum. Its NMR data were similar to aspergilliamide [21], which had been obtained previously. The ^1^H NMR data (Table 3) showed signals for two methylene groups at *δ*_H_ 2.49 (2H, d, *J* = 7.3 Hz, H-3′) and 2.37 (2H, d, *J* = 7.1 Hz, H-3); two methine groups at *δ*_H_ 2.13 (1H, m, H-4′) and 2.03 (1H, m, H-4); and four methyl groups at *δ*_H_ 1.04 (6H, d, *J* = 6.7 Hz, H-5′, 6′), 0.94 (6H, d, *J* = 6.7 Hz, H-5, 6), and ^13^C NMR data at *δ*_C_ 41.2 (C-3′), 33.1 (C-3), 26.9 (C-4′), 27.5 (C-4), 22.6 (C-5′,6′), 23.0 (C-5,6) suggested that **7** contained two isobutyl groups. In the HMBC spectrum (Figure 2), correlations from H-3′ to C-2′ (*δ*_C_ 172.0)/C-4′/C-5′/C-6′ suggested the existence of 3-methylbutanamide unit. This deduction was further verified by HMBC correlations from H-3 to C-1 (*δ*_C_ 164.5)/C-2 (*δ*_C_ 153.4) /C-4/C-5/C-6. The hydrolysis of **7** to give N-hydroxy-2-(hydroxyimino)-4-methylpentanamide (**8**) suggested the presence of **8** unit in **7** [21]. Based on the above data, the structure of **7** was deduced as N-hydroxy-2-(hydroxyimino)-4-methyl-N-(3-methylbutanoyl) pentanamide (**7**).

Compound **8** as yellowish powder was determined as an alkaline hydrolysate of **7**. The HRESIMS peak of [M − H]^−^ at *m/z* 159.0782 and NMR spectra of **8** confirmed its molecular formula as C_6_H_11_N_2_O_3_. The ^1^H NMR data (Table 3) showed signals for three exchangeable protons at *δ*_H_ 11.44 (1H, s, 2=N-OH), 10.68 (1H, brs, 1′-OH) and 8.90 (1H, brs, 1′-H)m and the remainder of the nine protons at *δ*_H_ 2.37 (2H, d, *J* = 7.3 Hz, H-3), 1.90 (1H, m, H-4), 0.85 (6H, d, *J* = 6.7 Hz, H-5, 6) were similar to those of **7** and ^13^C NMR data (Table 3) at *δ*_C_ 32.3 (C-3), 25.7 (C-4), 22.6 (C-5,6), which were suggested to also contain the isobutyl group. In the HMBC spectrum, correlations from H-3 to C-1 (*δ*_C_ 162.0)/C-2 (*δ*_C_ 153.1)/C-4/C-5/C-6 suggested the existence of 3-methylbutanamide unit. This deduction was further confirmed by HMBC correlations from H-1′ (*δ*_H_ 11.4, s) to C-2. Thus, the structure of **8** was identified N-hydroxy-2-(hydroxyimino)-4-methylpentanamide (**8**), which is suggested to be a hydrolytic product of **7**.

Compound **9** was obtained as a white powder, which had the molecular formula of C_6_H_12_N_2_O_2_ as inferred from HRESIMS (*m/z* 167.0791 [M + Na]^+^) and NMR data (Table 3). It had been afforded previously as a synthetic compound by the alkaline hydrolysis of aspergilliamide [21], and spectroscopic data were not reported. Thus, compound **9** was identified as (*E*)-2-(hydroxyimino)-4-methylpentanamide (**9**), and it is reported as a new natural product.

Compounds **10** and **11** were identified as Cyclo-(Ala-Leu) [22] and Cyclo-(Pro-Leu) [23] by comparing spectroscopic data with those reported in the literature.

### 2.2. Bioassays

As a conventional activity screening, compounds **1**–**9** were tested for their antibacterial (against *Acinetobacter baumannii*, *Staphylococcus aureus*, *Enterococcus faecalis*, *Klebsiella pneumoniae*, *S. aureus* subsp. aureus Rosenbach, methicillin-resistant *S. aureus*, and methicillin-resistant *S. epidermidis*) and cytotoxic (against human cancer cell lines HeLa, HCT-116, and A549) activities. However, none of them displayed obvious antibacterial or cytotoxicity activities.

The aromatic analogues containing a linear chain were reported with lowering lipid effects [24]. For example, 2-methyl-8-hydroxybenzeneheptanoic acid, an aromatic analogue obtained from a marine-derived *Streptomyces* strain, remarkably decreased lipid levels including total cholesterol (TC) and triglycerides (TG) in HepG2 cells [24]. Moreover, it is revealed that the aromatic analogue containing a linear chain with seven carbons showed stronger inhibition in comparison with those bearing a linear chain with five carbons [24]. In this study, several aromatic analogues containing a linear chain with eleven or thirteen carbons have been obtained, but their lipid-lowering effect remains unknown. Thus, the obtained new compounds, including the aromatic analogues, were evaluated with their effects on intracellular lipid accumulation in HepG2 cells by oil-red O staining, together with their brief SAR discussion.

**Figure 4:**
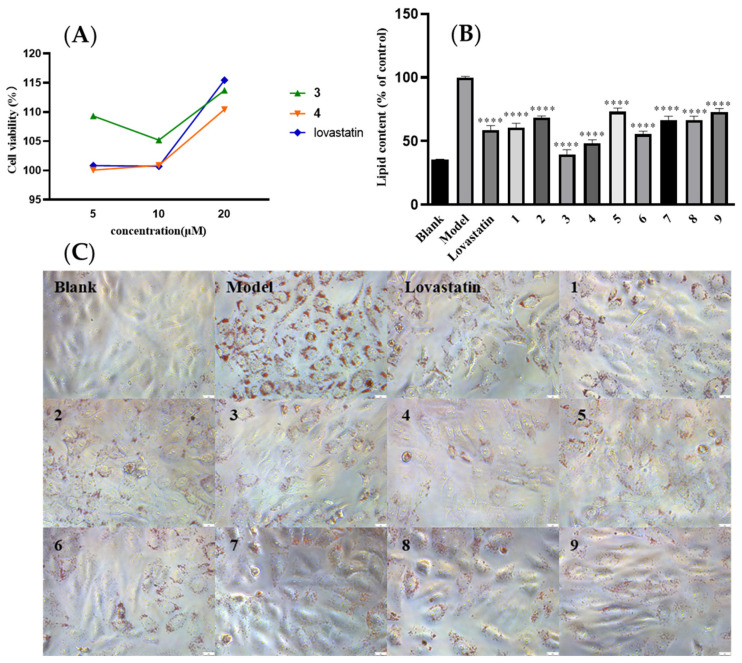
Effects of **1**–**9** on oleic acid-elicited intracellular lipid accumulation. (**A**) Cell viability of **3**, **4**, and lovastatin in HepG2 cells; (**B**) HepG2 cells were treated with isopropanol and intracellular lipid levels were measured using the absorbance at OD 510 nm; (**C**) Oil Red O staining showed lipid accumulation that was observed with a microscope (scale bar = 1 mm). Data were represented as the mean ± SEM of three in dependent experiments. **** *p* < 0.001, test group vs. model group.

The compounds **1**–**9** and the positive control lovastatin were tested for cytotoxic effects toward HepG2 cells by using the MTT (Sigma Aldrich) assay, together with oleic acid (OA). The results showed there is no obvious toxicity with these compounds. HepG2 cells were incubated with OA (500 μM) and DMEM for 24 h and then treated with 10 μM of indicated compounds or lovastatin for an additional 24 h. The effects of compounds **1**–**9** on oleic acid-elicited intracellular lipid accumulation were showed in Figure 4. All compounds tested showed a reduction in intracellular lipid accumulation, while **3** and **4** displayed stronger inhibition than the others. As for the results, there is no clear conclusion about the difference in activities in comparison with those aromatic analogues with eleven and thirteen carbons of the linear chain in the structures. However, it was speculated that the conjugated double bond in linear chain (such as in **3** or **4**) is beneficial for the intracellular lipid-lowering effect than carbonyl or hydroxyl groups in this position (Figure 4). The hypolipidemic mechanism of them needs further study.

## 3. Materials and Methods

### 3.1. General Experimental Procedures

Semipreparative HPLC (Agilent Technologies, 1260 Infinity II series) was performed using an ODS column (YMC-pack ODS-A, 10 mm × 250 mm, 5 μm). Column chromatography (CC) was performed over silica gel (200–300 mesh; Qingdao Marine Chemical Group Co., Qingdao, China) and Sephadex LH-20 (Amersham Biosciences Inc., Piscataway, NJ, USA) and octadecylsilyl silica gel (YMC Co., Ltd., Kyoto, Japan; 50 μm), respectively. Spots were detected on TLC (Qingdao Marine Chemical Factory, Qingdao, China) under 254 nm UV light. The NMR spectra were obtained on a Bruker Avance-600 MHz spectrometer (Bruker, Billerica, MA, USA) with tetramethylsilane as an internal standard. HR-ESI-MS spectra were recorded on a Bruker miXis TOF-QII mass spectrometer (Bruker, Billerica, MA, USA). Optical rotations were determined with a Perkin Elmer MPC 500 (Waltham, MA, USA) polarimeter. The UV, IR, and ECD spectra were recorded on a Shimadzu UV-2600 PC spectrometer (Shimadzu, Kyoto, Japan), an IR Affinity-1 spectrometer (Shimadzu, Kyoto, Japan), and a Chirascan circular dichroism spectrometer (Applied Photophysics, Leatherhead Surrey, UK), respectively. The artificial sea salt was a commercial product (Guangzhou Haili Aquarium Technology Company, Guangzhou, China). Cells were disrupted using a high pressure homogenizer NanoGenizer (Genizer LLC, Irvine, CA, USA).

### 3.2. Strain Material

The strain SCSIO15079 used in this investigation was isolated from the deep-sea sediment sample of the Indian Ocean (Lat: 10.00371667o N, long: 88.72803333o E) at a depth of 3386 m in 2013. It was identified as a *S. chumphonensis* species by 16S rRNA gene sequence analysis (Supplementary Material). The strain SCSIO15079 is preserved at the CAS Key Laboratory of Tropical Marine Bio-Resources and Ecology, South China Sea Institute of Oceanology, Chinese Academy of Sciences.

### 3.3. Cultivation and Extraction

A few loops of cells of the strain were inoculated into a 500 mL Erlenmeyer flask containing 150 mL of seed medium (malt extract 1%, yeast extract 0.4%, glucose 0.4%, pH 7.2) and then cultivated on a rotary shaker at 180 rpm, 28 °C for 48 h as seed culture. Then, 2 L of seed culture was inoculated into a 65 L fermenter containing 40 L medium (soybean meal 1%, corn steep liquor 0.5%, glucose 0.5%, yeast extract 0.1%, glycerol 2%, meat extract 0.2%, CaCO3 0.2%, MgSO_4_ 0.01%, pH7.2). After cultivation at 180 rpm and 28 °C for 80 h, the bacterial culture broth was centrifuged at 4000 rpm. Then, the mycelium and supernatant were broken using an ultrasonic treatment apparatus for 15 min and extracted three times with an equal volume of ethyl acetate, respectively. The organic extract was then concentrated under a vacuum to provide the crude extract. EtOAc was concentrated in vacuo to yield an organic extract (about 51 g).

### 3.4. Isolation and Purification

The EtOAc crude extract (51 g) was subjected to silica gel vacuum liquid chromatography using a step gradient elution of petroleum ether (PE)−EtOA (0–100%, *V/V*), EtOAc−MeOH (0–100%, *V/V*) to yield nine fractions according to TLC profiles (Fr.1–Fr.8). Fr. 2 (1.2 g) was separated into four subfractions (Fr. 2.1–2.4) by silica gel chromatography eluting with CH_2_Cl_2_/MeOH (0–100%, *V/V*), and Fr. 2.2 was directly separated by semipreparative HPLC (85% MeCN/H_2_O, 4 mL/min, 210 nm) to yield **1** (6.5 mg, t_R_ 26.7 min) and **2** (21.3 mg, t_R_ 36.2 min). From Fr. 3 (2.3 g), **3** (11.6 mg, t_R_ 29.0 min) and **4** (41.0 mg, t_R_ 58.3 min) were repurified through a silica gel CC with PE/EtOA (10–100%, *V/V*) and semipreparative HPLC (73% MeCN/H_2_O, 3 mL/min, 254 nm). The subsequent purification of Fr. 6 (1.5 g) using Sephadex LH-20 with MeOH and semipreparative HPLC (70% MeOH/H_2_O, 3 mL/min, 210 nm) afforded **5** (39 mg, t_R_ 23.5 min) and **6** (9.2 mg, t_R_ 34 min). Fr. 5 (1.6 g) was eluted with MeOH-H2O (1:9 to 1:0) on ODS CC and further purified on HPLC with MeOH–H_2_O gradient (5:95 to 100:0, 3 mL/min, total 40 min, 210 nm) to give **7** (10.2 mg, t_R_ 24.5 min) and **8** (8.0 mg, t_R_ 32.3 min). Fr. 4 (3.6 g) was further separated to seven subfractions (Fr. 4.1–4.8) by ODS silica gel CC eluted with MeOH-H_2_O gradient (1:9 to 1:0). Fr. 4.3 (563 mg) was subjected to CC on Sephadex LH-20 eluting with MeOH and then further purified by semi-preparative HPLC (MeOH–H2O 3:7, 3 mL/min, 210 nm) to afford **9** (t_R_ = 8.6 mg, 30.2 min). Fr. 4.7 (135 mg) was further purified on semipreparative HPLC (20% MeCN/H_2_O, 3 mL/min, 210 nm) to obtain **10** (3.4 mg, t_R_ 24.0 min) and **11** (5.2 mg, t_R_ 33.2 min).

11-Oxo-11-(o-tolyl) undecanoic acid (**1**): yellowish solid; UV (MeOH) λ_max_ (log ε) 206 (2.30), 241 (2.37), 279 (2.43) and 323 (2.50) nm; IR (film) *ν*_max_ 2926, 2852, 1732, 1681, 1506, 1465 1226, 1183, 966, 742, 653 cm^−1^; ^1^H and ^13^C NMR (see Table 1 and Table 2); and HR-ESIMS *m/z* 289.1809, [M − H]^−^ (calculated for C_18_H_25_O_3_ 289.1804).

13-Oxo-13-(o-tolyl) tridecanoic acid (**2**): yellowish solid; UV (MeOH) λ_max_ (log ε) 206 (2.34), 241 (2.41) and 283 (2.47) nm; IR (film) *ν*_max_ 2920, 2852, 1705, 1681, 1543, 1319 1219, 1182, 962, 759, 655 cm^−1^; ^1^H and ^13^C NMR (see Table 1 and Table 2); and HR-ESIMS *m/z* 319.2268, [M + H]^+^ (calculated for C_20_H_31_O_3_ 319.2273).

(*E*)-11-(o-tolyl) Undec-10-enoic acid (**3**): pale yellow oil; UV (MeOH) λ_max_ (log ε) 209 (2.31), 249 (2.38), 287 (2.44), 298 (2.46) and 323 (2.50) nm; IR (film) *ν*_max_ 2920, 2860, 1738, 1680, 1553, 1450, 1281, 1183, 968, 760 cm^−1^; ^1^H and ^13^C NMR (see Table 1 and Table 2); and HR-ESIMS *m/z* 297.1825, [M+Na]^+^ (calculated for C_18_H_26_O_2_Na 297.1830).

(*E*)-13-(o-tolyl) Tridec-12-enoic acid (**4**): pale yellow solid; UV (MeOH) λ_max_ (log ε) 210 (2.35), 248 (2.42), 286 (2.48), 298 (2.50) and 323 (2.54) nm; IR (film) *ν*_max_ 2923, 2855, 1736, 1683, 1554, 1452, 1281, 1184, 966, 761 cm^−1^; ^1^H and ^13^C NMR (see Table 1 and Table 2); and HR-ESIMS *m/z* 325.2139, [M + Na]^+^ (calculated for C_20_H_30_O_2_Na 325.2147).

(*R*)-11-Hydroxy-11-(o-tolyl) undecanoic acid (**5**): yellow oil; [α]${}_{D}^{25}$ +4.0 (c 0.10, MeOH); UV (MeOH) λ_max_ (log ε) 210 (2.31), 264 (2.41), 323 (2.50) and 371 (2.56) nm; IR (film) *ν*_max_ 2926, 2854, 1734, 1676, 1541, 1456, 1280, 1182, 966, 761 cm^−1^; CD (0.25 mg/mL, MeOH) λ_max_ (∆ε) 210 (+0.051), 264 (−0.044), 323 (+0.025) and 370 (−0.042) nm; ^1^H and ^13^C NMR (see Table 1 and Table 2); and HR-ESIMS *m/z* 291.1969, [M − H]^−^ (calculated for C_18_H_27_O_3_ 291.1960).

(*R*)-13-Hydroxy-13-(o-tolyl) tridecanoic acid (**6**): pale yellow solid; [α]${}_{D}^{25}$ +2.6 (c 0.10, MeOH); UV (MeOH) λ _max_ (log ε) 210 (2.35), 262 (2.44), 323 (2.54) and 370 (2.60) nm; IR (film) *ν*_max_ 2920, 2852, 1732, 1677, 1543, 1458 1281, 1183, 964, 760 cm^−1^; ^1^H and ^13^C NMR (see Table 1 and Table 2); and HR-ESIMS *m/z* 319.2279, [M − H]^−^ (calculated for C_20_H_31_O_3_ 319.2273).

N-Hydroxy-2-(hydroxyimino)-4-methyl-N-(3-methylbutanoyl) pentanamide (**7**): yellowish powder; UV (MeOH) λ_max_ (log ε) 207 (2.23) and 323 (2.42) nm; IR (film) *ν*_max_ 3915, 2927, 2872, 1653, 1554, 1095, 1002, 896, 724 cm^−1^; ^1^H and ^13^C NMR (see Table 3); and HR-ESIMS *m/z* 245.1496, [M + H]^+^ (calculated for C_11_H_21_O_4_N_2_ 245.1501).

N-Hydroxy-2-(hydroxyimino)-4-methylpentanamide (**8**): yellowish powder; UV (MeOH) λ_max_ (log ε) 209 (2.05) and 323 (2.24) nm; IR (film) *ν*_max_ 3315, 2956, 2872, 1653, 1456 1095, 1002, 898, 725 cm^−1^; ^1^H and ^13^C NMR (see Table 3); and HR-ESIMS *m/z* 159.0775, [M − H]^−^ (calculated for C_6_H_11_O_3_N_2_ 159.0770).

2-(Hydroxyimino)-4-methylpentanamide (**9**): white powder; UV (MeOH) λ_max_ (log ε) 213 (2.01) and 323 (2.19) nm; IR (film) *ν*_max_ 3325, 1635, 1016, 667, 555 cm^−1^; ^1^H and ^13^C NMR (see Table 3); and HR-ESIMS *m/z* 67.0791, [M + Na]^+^ (calculated for C_6_H_12_O_2_N_2_Na 167.0796).

### 3.5. ECD Calculation

Conformational searches were performed by employing the “systematic” procedure in the Spartan’14 software using a Molecular Merck force field. The stable conformers were further chosen for ECD calculations by TD-DFT methodology via the Gaussian 16 program [25], which were calculated at the B3LYP/6-311+G (d, p)//B3LYP/6-31+G (d) level by adopting the polarizable continuum model and 30 excited states. The calculated ECD spectra were generated by the program SpecD is 1.71 using Gaussian band shapes from dipole length rotational strengths according to Boltzmann distributions.

### 3.6. Mono-MTPA Esters of **5**

Compounds **5** (1.3 mg) was dissolved in freshly distilled dry pyridine (200 μL) with dry crystals of dimethylaminopyridine (DMAP, 0.5 mg). The treatment with (*R*)-MTPA-Cl at 28 °C yielded the *S*-MTPA ester after 18 h. The reaction mixture was purified by semipreparative HPLC (70% CH_3_CN in H_2_O) to produce the *S*-MTPA ester after 30 min. The R-MTPA ester was prepared with *S*-MTPA-Cl in the same manner. The ∆δ *S*−*R* values for the mono-*S*- and *R*-MTPA esters of compounds **5** were recorded in ppm in CD_3_OD.

### 3.7. Antihyperlipidemic Effects

#### 3.7.1. Cell Culture and Determination of Oleic Acid Concentration

HepG2 cells were cultured in Dulbecco’s modified Eagle’s medium containing 10% fetal bovine serum and 1% penicillin–streptomycin in a humidified incubator with 5% CO_2_ at 37 °C, and cells in the logarithmic phase were taken for the experiments [26]. The oleic acid (OA) was prepared with 10% bovine serum albumin (BSA) and added to the culture medium at a final concentration of 1%, as previously described. Blank control cells were treated with 1% BSA. After reaching 70% confluence, HepG2 cells were serum-starved for18 h and exposed to 0.5 μM OA, with or without compound/positive, for 24 h [27].

#### 3.7.2. Cell Viability Assay

Cell viability was measured by an MTT assay. In brief, HepG2 cells were plated at a density of 2 × 10^4^ cells/well in 96-well plates. After the cells were attached to the plate, they were treated with the OA (0.25, 0.5, 1 mM) or compounds/lovastatin (5, 10, 20 µM). After 24 h, 20 µL of an MTT solution (5 mg/mL) was added to each well, followed by incubation for 4 h. The medium was removed, and the formazan crystals formed in living cells were dissolved in 150 µL of DMSO. Cell viability was measured at 490 nm using an iMak microplate reader (Molecular Devices, Bio-Rad, Hercules, CA, USA). Each treatment was performed in triplicate.

#### 3.7.3. Oil Red O Staining

Cells were fixed with 4% paraformaldehyde, and Oil Red O staining was performed [24]. Oil droplets were observed using microscopy (Olympus, Tokyo, Japan). Next, cells were treated with isopropanol and lipid accumulation were measured using a microplate reader and recording the absorbance at 510 nm [28].

### 3.8. Antimicrobial Assay

Compounds **1**–**9** were tested for antibacterial activities against seven bacterial strains: *Acinetobacter baumannii* (ATCC 19606), *Staphylococcus aureus* (ATCC 29213), *Enterococcus faecalis* (ATCC 29212), *Klebsiella pneumoniae* (ATCC 13883), *S. aureus* subsp. aureus Rosenbach (ATCC 43300), methicillin-resistant *S. aureus* (MRSA, clinical strain), and methicillin-resistant *S. epidermidis* (MRSE, clinical strain), respectively; they were were evaluated in 96-well plates using a modification of the broth microdilution method [29].

### 3.9. Cell Culture and Cytotoxic Bioassay

Human cancer cell lines, HeLa, HCT-116, and A549, were purchased from ATCC. The HCT-116 cells were grown and maintained in a RPMI-1640 medium with 10% FBS, while the other cells were grown in a DMEM medium with 10% FBS. The cell viability was determined using a MTT assay with 50 μM of compounds **1**–**9**.

## 4. Conclusions

In this study, nine new compounds, including six aromatic analogues (**1**–**6**) and three leucine derivatives containing an unusual oxime moiety (**7**–**9**), were obtained from the deep-sea-derived actinomycete strain *Streptomyces chumphonensis* SCSIO15079. The new structures including absolute configurations were determined by spectroscopic methods coupled with experimental and calculated ECD. All of those new compounds showed inhibitory activities with intracellular lipid accumulation in HepG2 cells, while compound **3** and **4**, with a conjugated double bond in linear chain, displayed stronger inhibition than others. It will provide a new type of potential lead compounds for the development of antihyperlipidemic therapeutics.

## Figures and Tables

**Table 1:** ^1^H NMR (600 MHz) data of compounds **1**–**6** in CD_3_OD (*δ* in ppm).

Position	1 (*J* in Hz)	2 (*J* in Hz)	3 (*J* in Hz)	4 (*J* in Hz)	5 (*J* in Hz)	6 (*J* in Hz)
2	2.26 (t, 7.1)	2.27 (t, 7.3)	2.24 m	2.25 m	2.27 m	2.25 m
3	1.59 m	1.58 m	1.59 m	1.58 m	1.58 m	1.58 m
4	1.28~1.34 m	1.25~1.36 m	1.27~1.33 m	1.28~1.31 m	1.27~1.35 m	1.23~1.34 m
5						
6						
7						
8						
9	1.66 m		2.24 m		1.45 m	
10	2.91 (t, 7.3)		6.06 (dt, 7.02, 15.6)		1.70 m	
11		1.65 m	6.57 (d, 15.6)	2.24 m	4.86 m	1.46 m
12		2.90 (t, 7.3)		6.07 (dt, 7.0, 15.7)		1.67 m
13				6.57 (d, 15.7)		4.86 (dd, 4.3, 7.6)
1′						
2′						
3′	7.38 m	7.37 m	7.07 m	7.07 m	7.17 m	7.16 m
4′	7.27 m	7.27 m			7.10 (d, 4.1)	7.10 (d, 4.3)
5′						
6′	7.67 (d, 7.6)	7.66 (d, 7.7)	7.35 (d, 7.3)	7.35 (d, 7.3)	7.41 (d, 7.7)	7.40 (d, 7.6)
7′	2.42 s	2.42 s	2.28 s	2.28 s	2.32 s	2.31 s

**Table 2:** ^13^C NMR (150 MHz) data of compounds **1**–**6** in CD_3_OD (*δ* in ppm).

Position	1	2	3	4	5	6
1	178.2 C	178.1 C	178.6 C	177.9 C	177.9 C	177.9 C
2	35.3 CH_2_	35.2 CH_2_	34.3 CH_2_	34.4 CH_2_	35.0 CH_2_	35.0 CH_2_
3	25.6 CH_2_	25.6 CH_2_	26.3 CH_2_	26.1 CH_2_	26.1 CH_2_	26.0 CH_2_
4	30.3 CH_2_	30.3 CH_2_	30.3 CH_2_	30.3 CH_2_	30.4 CH_2_	30.2 CH_2_
5	30.2 CH_2_	30.2 CH_2_	30.2 CH_2_	30.2 CH_2_	30.2 CH_2_	30.1 CH_2_
6	30.3 CH_2_	30.4 CH_2_	30.4 CH_2_	30.4 CH_2_	30.5 CH_2_	30.4 CH_2_
7	30.4 CH_2_	30.4 CH_2_	30.5 CH_2_	30.5 CH_2_	30.6 CH_2_	30.5 CH_2_
8	30.5 CH_2_	30.5 CH_2_	30.6 CH_2_	30.7 CH_2_	30.6 CH_2_	30.5 CH_2_
9	26.2 CH_2_	30.6 CH_2_	34.3 CH_2_	30.8CH_2_	27.0 CH_2_	30.5 CH_2_
10	42.6 CH_2_	30.6 CH_2_	133.2 CH	30.8 CH_2_	39.3 CH_2_	30.5 CH_2_
11	207.4 C	26.2 CH_2_	129.0 CH	35.09 CH_2_	71.1 CH	26.9 CH_2_
12		42.6 CH_2_		133.2 CH		39.2 CH_2_
13		207.5 C		129.0 CH		71.0 CH
1′	139.7 C	139.7 C	138.2 C	138.2 C	144.6 C	144.4 C
2′	138.6 C	138.6 C	135.8 C	135.8 C	135.5 C	135.3 C
3′	132.8 CH	132.8 CH	127.0 CH	127.0 CH	127.1 CH	126.8 CH
4′	126.9 CH	126.9 CH	131.0 CH	131.1 CH	131.2 CH	131.0 CH
5′	132.3 CH	132.3 CH	127.8 CH	127.8 CH	127.8 CH	127.7 CH
6′	129.5 CH	129.5 CH	126.4 CH	126.5 CH	126.4 CH	126.3 CH
7′	21.1 CH_3_	21.2 CH_3_	19.9 CH_3_	19.9 CH_3_	19.3 CH_3_	19.2 CH_3_

**Table 3:** ^1^H and ^13^C NMR data (600/150 MHz) of compounds **7**–**9** (*δ* in ppm).

Position	7 ^a^		8 ^b^		9 ^a^	
	*δ*_C_	*δ*_H_ (*J* in Hz)	*δ*_C_	*δ*_H_ (*J* in Hz)	*δ*_C_	*δ*_H_ (*J* in Hz)
1	164.5 C		162.0 C		168.9 C	
2=N-OH	153.4 C		153.1 C	11.4 s	154.3 C	
3	33.1 CH_2_	2.37 (d, 7.1)	32.3 CH_2_	2.37 (d, 7.3)	32.6 CH_2_	2.48 (d,7.3)
4	27.5 CH	2.03 m	25.7 CH	1.90 m	27.6 CH	2.01 m
5	23.0 CH_3_	0.94 (d, 6.7)	22.6 CH_3_	0.85 (d, 6.7)	23.1 CH_3_	0.92 (d, 6.7)
6	23.0 CH_3_	0.94 (d, 6.7)	22.6 CH_3_	0.85 (d, 6.7)	23.1 CH_3_	0.92 (d, 6.7)
1′-H				8.9 br.s		
1′-OH				10.7 br.s		
2′	172.0 C					
3′	41.2 CH_2_	2.49 (d, 7.3)				
4′	26.9 CH	2.13 m				
5′	22.6 CH_3_	1.04 (d, 6.7)				
6′	22.6 CH_3_	1.04 (d, 6.7)				

^a^ Data measured in CD_3_OD; ^b^ data measured in DMSO-*d*_6_.

## Supplementary Materials

The following supporting information can be downloaded at: https://www.mdpi.com/article/10.3390/md20040259/s1, 16S rRNA Sequence of the Strain SCSIO15079; Figures S1–S67: ^1^H NMR, ^13^C NMR, DEPT, HSQC, HMBC, COSY, NOESY, HRESIMS, IR, and UV spectra of compounds **1**–**9**; Figure S68. Hydrolysis reaction of **7**; Table S1. Cell viability of HepG2 treated with compounds.
