# Supplementary material for: Aromatic Acids and Leucine Derivatives Produced from the Deep-Sea Actinomycetes Streptomyces chumphonensis SCSIO15079 with Antihyperlipidemic Activities
Source: Mar Drugs. 2022 Apr 7;20(4):259. doi: 10.3390/md20040259 (PMC9026450; doi:10.3390/md20040259)
Supplement: Supplementary file 1 [file marinedrugs-20-00259-s001.zip › marinedrugs-1651371-supplementary.pdf]

Supplementary Materials

Aromatic Acids and Leucine Derivatives Produced from the Deep-Sea Actinomycetes *Streptomyces Chumphonensis* SCSIO15079 with Antihyperlipidemic Activities

Ziqi Su [1], Kunlong Li [2,3], Xiaowei Luo [4], Yongyan Zhu [1], Shaoyu Mai [1], Quanhong Zhu [1], Bin Yang [2,3], Xuefeng Zhou [2, 3,]* and Huaming Tao [1,]*

1 Guangdong Provincial Key Laboratory of Chinese Medicine Pharmaceutics, School of Traditional Chinese Medicine, Southern Medical University, Guangzhou 510515; ziqisl@163.com (Z.S.); yong-yanzhu0521@163.com (Y.Z.); shaoyumai@smu.edu.cn (S.M.); ; zqh@smu.edu.cn (Q.Z.)

2 CAS Key Laboratory of Tropical Marine Bio-Resources and Ecology, Guangdong Key Laboratory of Marine Materia Medica, South China Sea Institute of Oceanology, Chinese Academy of Sciences, Guangzhou 510301, China; likunlong16@mails.ucas.ac.cn (K.L.); yangbin@scsio.ac.cn (B.Y.)

3 Southern Marine Science and Engineering Guangdong Laboratory (Guangzhou), Guangzhou 511458, China

4 Institute of Marine Drugs, Guangxi University of Chinese Medicine, Nanning 530200, China; luoxiao-wei1991@126.com (X.L.)

* Correspondence: xfzhou@scsio.ac.cn (X.Z.); taohm@smu.edu.cn (H.T.); Tel.: +86-61648770 (H.T.)

List of Supplementary Materials

16S rRNA Sequence of the Strain SCSIO15079

Figure S1. ^{1}H NMR spectrum of compound **1** (CD_3OD, 600 MHz)

Figure S2. ^{13}C NMR spectrum of compound **1** (CD_3OD, 150 MHz)

Figure S3. ^{1}H-^{1}HCOSY spectrum of compound **1** (CD_3OD)

Figure S4. HSQC spectrum of compound **1** (CD_3OD)

Figure S5. HMBC spectrum of compound **1** (CD_3OD)

Figure S6. HRESIMS spectrum of compound **1**

Figure S7. IR spectrum of compound **1**

Figure S8. UV spectrum of compound **1**

Figure S9. ^{1}H NMR spectrum of compound **2** (CD_3OD, 600 MHz)

Figure S10. ^{13}C NMR spectrum of compound **2** (CD_3OD, 150 MHz)

Figure S11. ^{1}H-^{1}HCOSY spectrum of compound **2** (CD_3OD)

Figure S12. HSQC spectrum of compound **2** (CD_3OD)

Figure S13. HMBC spectrum of compound **2** (CD_3OD)

Figure S14. HRESIMS spectrum of compound **2**

Figure S15. IR spectrum of compound **2**

Figure S16. UV spectrum of compound **2**

Figure S17. ^{1}H NMR spectrum of compound **3** (CD_3OD, 600 MHz)

Figure S18. ^{13}C NMR spectrum of compound **3** (CD_3OD, 150 MHz)

Figure S19. ^{1}H-^{1}HCOSY spectrum of compound **3** (CD_3OD)

Figure S20. HSQC spectrum of compound **3** (CD_3OD)

Figure S21. HMBC spectrum of compound **3** (CD_3OD)

Figure S22. HRESIMS spectrum of compound **3**

Figure S23. UV spectrum of compound **3**

Figure S24. ^{1}H NMR spectrum of compound **4** (CD_3OD, 600 MHz)

Figure S25. ^{13}C NMR spectrum of compound **4** (CD_3OD, 150 MHz)

Figure S26. ^{1}H-^{1}HCOSY spectrum of compound **4** (CD_3OD)

Figure S27. HSQC spectrum of compound **4** (CD_3OD)

Figure S28. HMBC spectrum of compound **4** (CD_3OD)

Figure S29. HRESIMS spectrum of compound **4**

Figure S30. UV spectrum of compound **4**

Figure S31. ^{1}H NMR spectrum of compound **5** (CD_3OD, 600 MHz)

Figure S32. ^{13}C NMR spectrum of compound **5** (CD_3OD, 150 MHz)

Figure S33. ^{1}H-^{1}HCOSY spectrum of compound **5** (CD_3OD)

Figure S34. HSQC spectrum of compound **5** (CD_3OD)

Figure S35. HMBC spectrum of compound **5** (CD_3OD)

Figure S36. HRESIMS spectrum of compound **5**

Figure S37. UV spectrum of compound **5**

Figure S38. ^{1}H NMR spectrum of compound **6** (CD_3OD, 600 MHz)

Figure S39. ^{13}C NMR spectrum of compound **6** (CD_3OD, 150 MHz)

Figure S40. ^{1}H-^{1}HCOSY spectrum of compound **6** (CD_3OD)

Figure S41. HSQC spectrum of compound **6** (CD_3OD)

Figure S42. HMBC spectrum of compound **6** (CD_3OD)

Figure S43. HRESIMS spectrum of compound **6**

Figure S44. UV spectrum of compound **6**

Figure S45. ^{1}H NMR spectrum of compound **7** (CD_3OD, 600 MHz)

Figure S46. ^{13}C NMR spectrum of compound **7** (CD_3OD, 150 MHz)

Figure S47. ^{1}H-^{1}HCOSY spectrum of compound **7** (CD_3OD)

Figure S48. HSQC spectrum of compound **7** (CD_3OD)

Figure S49. HMBC spectrum of compound **7** (CD_3OD)

Figure S50. HRESIMS spectrum of compound **7**

Figure S51. UV spectrum of compound **7**

Figure S52. ^1H NMR spectrum of compound **8** (MDSO-d_6, 600 MHz)

Figure S53. ^{13}C NMR spectrum of compound **8** (MDSO-d_6, 150 MHz)

Figure S54. ^1H-^1HCOSY spectrum of compound **8** (MDSO-d_6)

Figure S55. HSQC spectrum of compound **8** (MDSO-d_6)

Figure S56. HMBC spectrum of compound **8** (MDSO-d_6)

Figure S57. HRESIMS spectrum of compound **8**

Figure S58. IR spectrum of compound **8**

Figure S59. UV spectrum of compound **8**

Figure S60. ^1H NMR spectrum of compound **9** (CD_3OD, 600 MHz)

Figure S61. ^{13}C NMR spectrum of compound **9** (CD_3OD, 150 MHz)

Figure S62. ^1H-^1HCOSY spectrum of compound **9** (CD_3OD)

Figure S63. HSQC spectrum of compound **9** (CD_3OD)

Figure S64. HMBC spectrum of compound **9** (CD_3OD)

Figure S65. HRESIMS spectrum of compound **9**

Figure S66. IR spectrum of compound **9**

Figure S67. UV spectrum of compound **9**

Figure S68. Hydrolysis reaction of compound **7**

Table S1. Cell Viability (%) of HepG2 treated with compounds **1-9** and lovastatin (5, 10, and 20 μM).

16S rRNA Sequence of the Strain SCSIO15079

CGCTTCGGTGGTGGATTAGTGGCGAACGGGTGAGTAACACGTGGGCAATCTGCCCTG
CACTCTGGGACAAGCCCTGGAAACGGGGTCTAATACCGGATACGACACAGGAAGGC
ATCTTCTCTGTGTGGAAAGCTCCGGCGGTGCAGGATGAGCCCGCGGCCTATCAGCTTG
TTGGTGGGGTGATGGCCTACCAAGGCGACGACGGGTAGCCGGCCTGAGAGGGCGACC
GGCCACACTGGGACTGAGACACGGCCCAGACTCCTACGGGAGGCAGCAGTGGGGAA
TATTGCACAATGGGCGAAAGCCTGATGCAGCGACGCCGCGTGAGGGATGACGGCCTT
CGGGTTGTAAACCTCTTTCAGCAGGGAAGAAGCGCAAGTGACGGTACCTGCAGAAGA
AGCACCGGCTAACTACGTGCCAGCAGCCGCGGTAATACGTAGGGTGCGAGCGTTGTC
CGGAATTATTGGGCGTAAAGAGCTCGTAGGCGGCTTGTCACGTCGGATGTGAAAGCC
CGGGGCTTAACCCCGGGTCTGCATTCGATACGGGCAGGCTAGAGTTCGGTAGGGGAG
ATCGGAATTCCTGGTGTAGCGGTGAAATGCGCAGATATCAGGAGGAACACCGGTGGC
GAAGGCGGATCTCTGGGCCGATACTGACGCTGAGGAGCGAAAGCGTGGGGAGCGAA
CAGGATTAGATACCCTGGTAGTCCACGCCGTAAACGTTGGGAACTAGGTGTGGGCGA
CATTCCACGTCGTCCGTGCCGCAGCTAACGCATTAAGTTCCCCGCCTGGGGAGTACGG
CCGCAAGGCTAAAACTCAAAGGAATTGACGGGGGCCCGCACAAGCGGCGGAGCATG
TGGCTTAATTCGACGCAACGCGAAGAACCTTACCAAGGCTTGACATACATCGGAAAG
CCGTAGAGATACGGCCCCCCTTGTGGTCGGTGTACAGGTGGTGCATGGCTGTCGTCAG
CTCGTGTCGTGAGATGTTGGGTTAAGTCCCGCAACGAGCGCAACCCTTATTCTGTGTT
GCCAGCATGCCTTTCGGGGTGATGGGGACTCACAGGAGACTGCCGGGGTCAACTCGG
AGGAAGGTGGGGACGACGTCAAGTCATCATGCCCCTTATGTCTTGGGCTGCACACGT
GCTACAATGGCCGGTACAATGAGCTGCGATACCGTGAGGTGGAGCGAATCTCAAAAA
GCCGGTCTCAGTTCGGATTGGGGTCTGCAACTCGACCCCATGAAGTCGGAGTCGCTA
GTAATCGCAGATCAGCATTGCTGCGGTGAATACGTTCCCGGGCCTTGTACACACCGCC
CGTCACGTCACGAAAGTCGGTAACACCCGAAGCCGGTGGCCTAACCCC

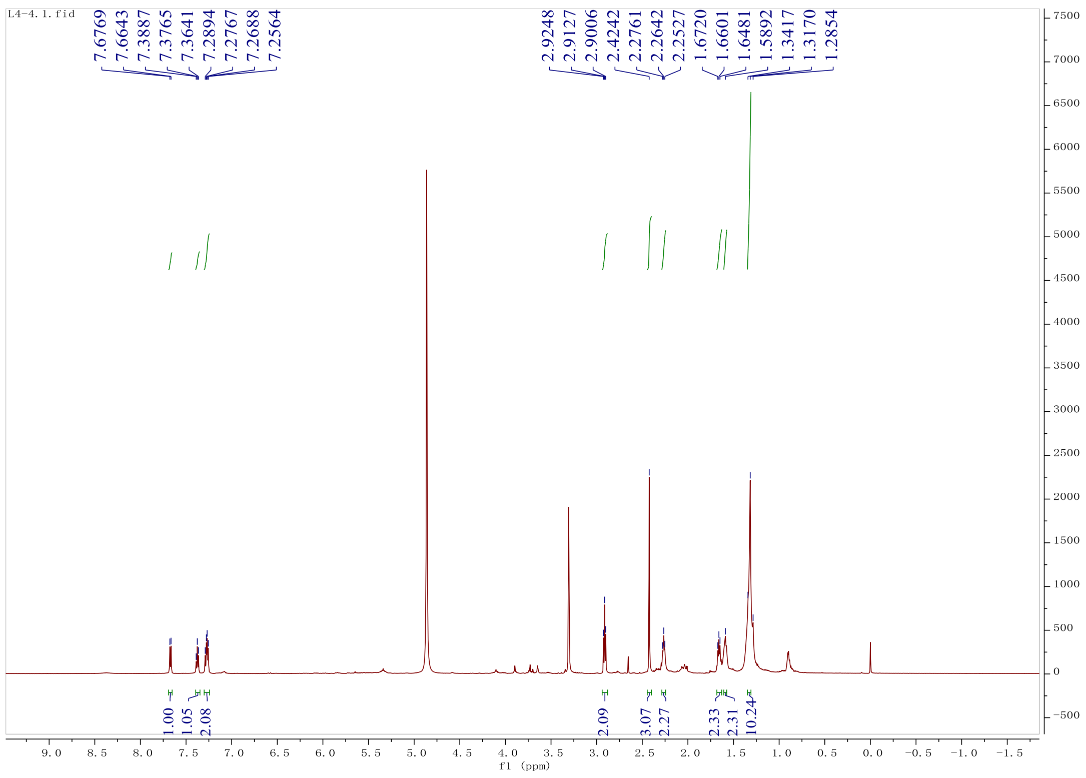

Figure S1. ^{1}H NMR spectrum of compound **1** (CD_3OD, 600 MHz)

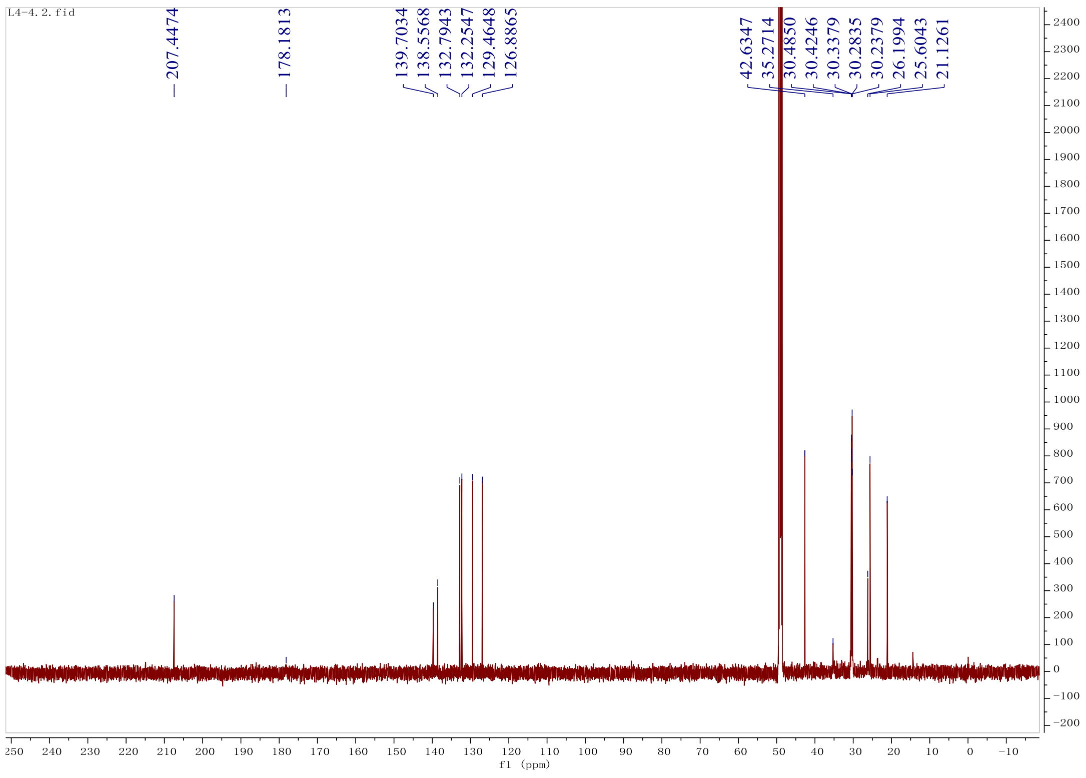

Figure S2. ^{13}C NMR spectrum of compound **1** (CD_3OD, 150 MHz)

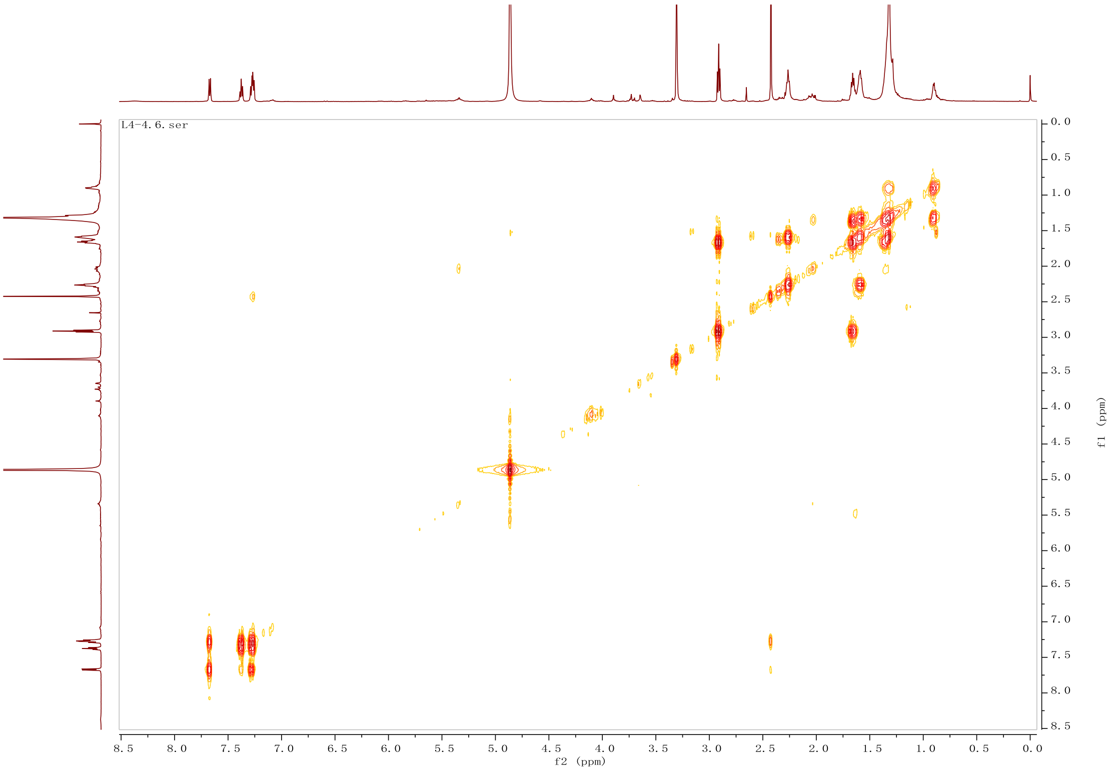

Figure S3. ^{1}H-^{1}HCOSY spectrum of compound **1** (CD_3OD)

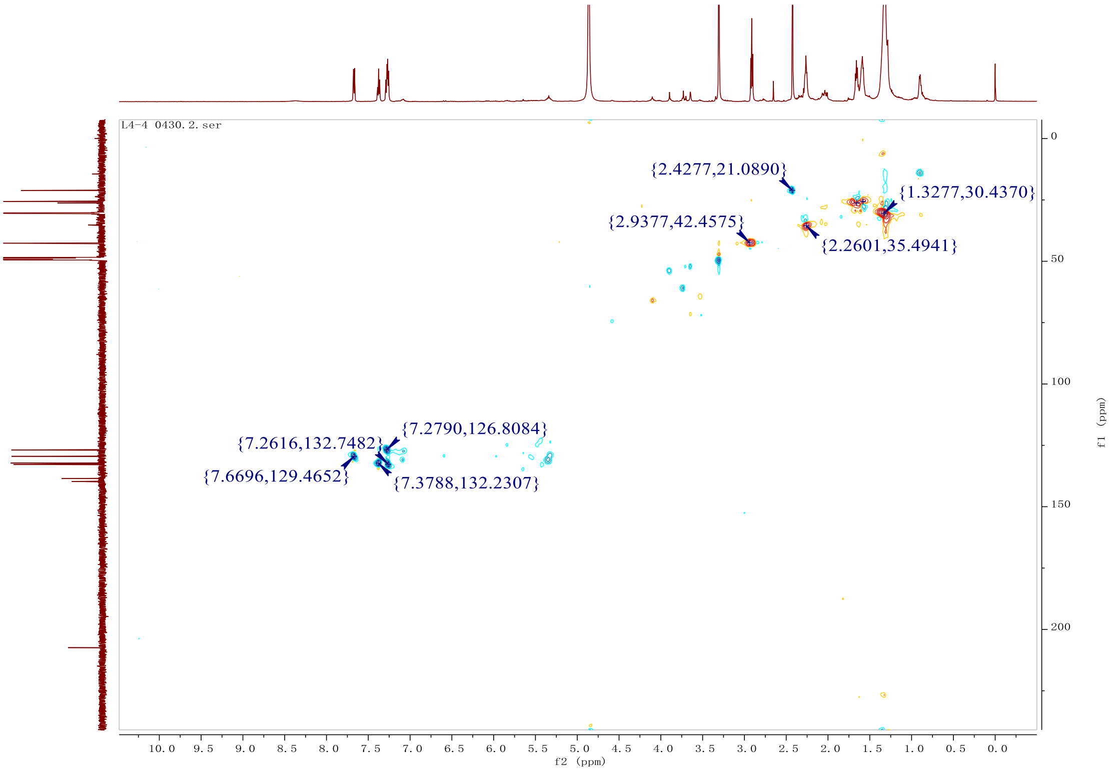

Figure S4. HSQC spectrum of compound **1** (CD_3OD)

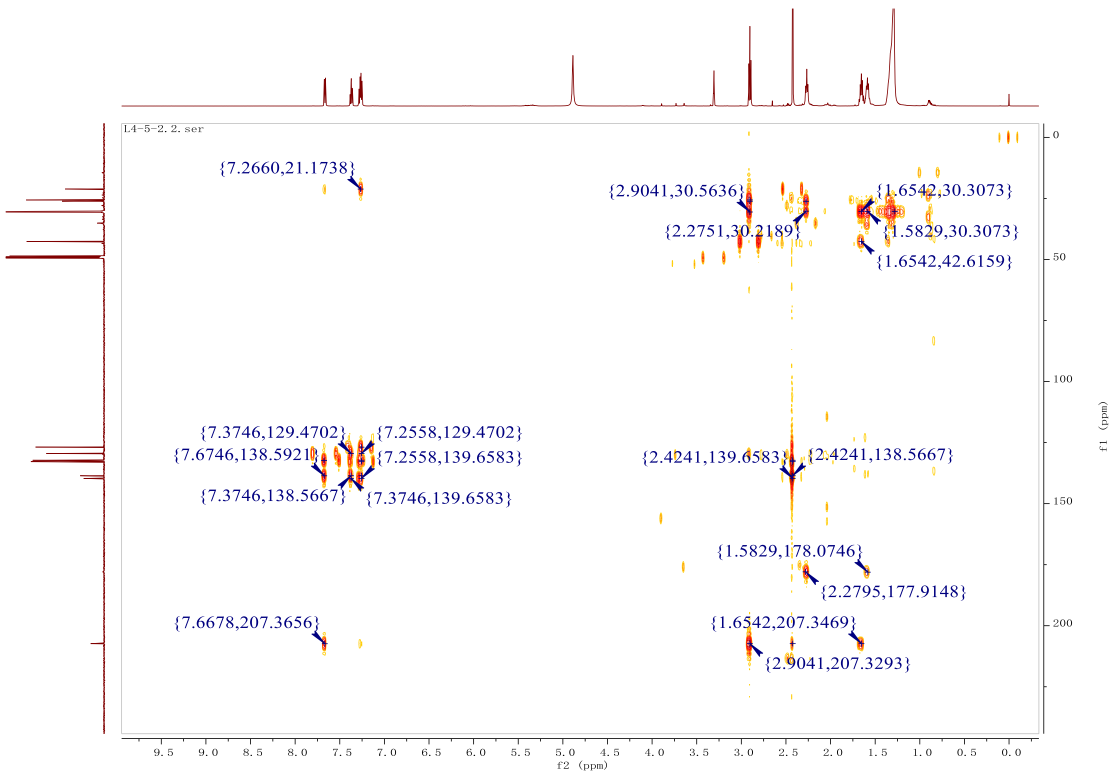

Figure S5. HMBC spectrum of compound **1** (CD_3OD)

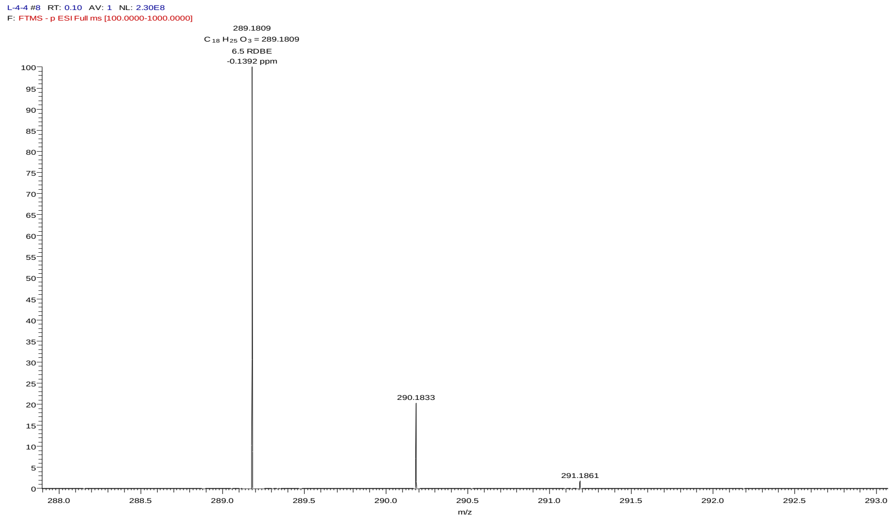

Figure S6. HRESIMS spectrum of compound **1**

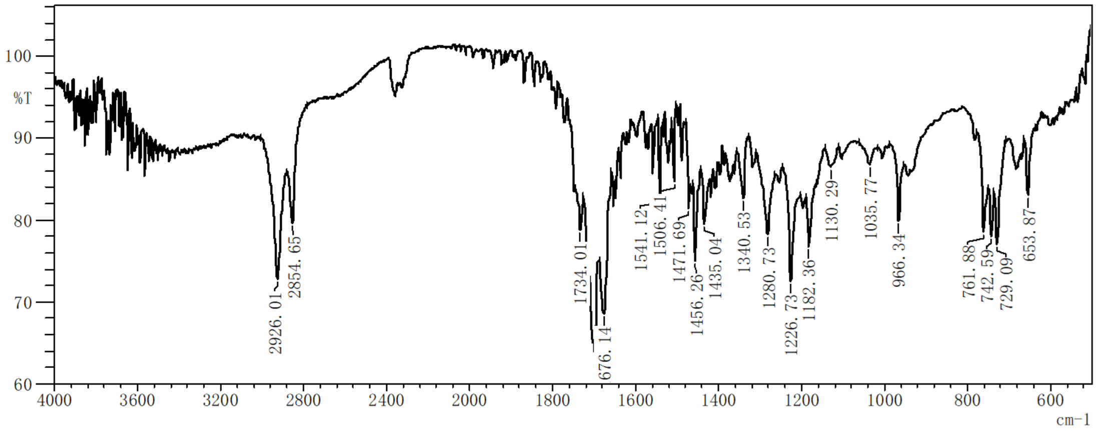

Figure S7. IR spectrum of compound **1**

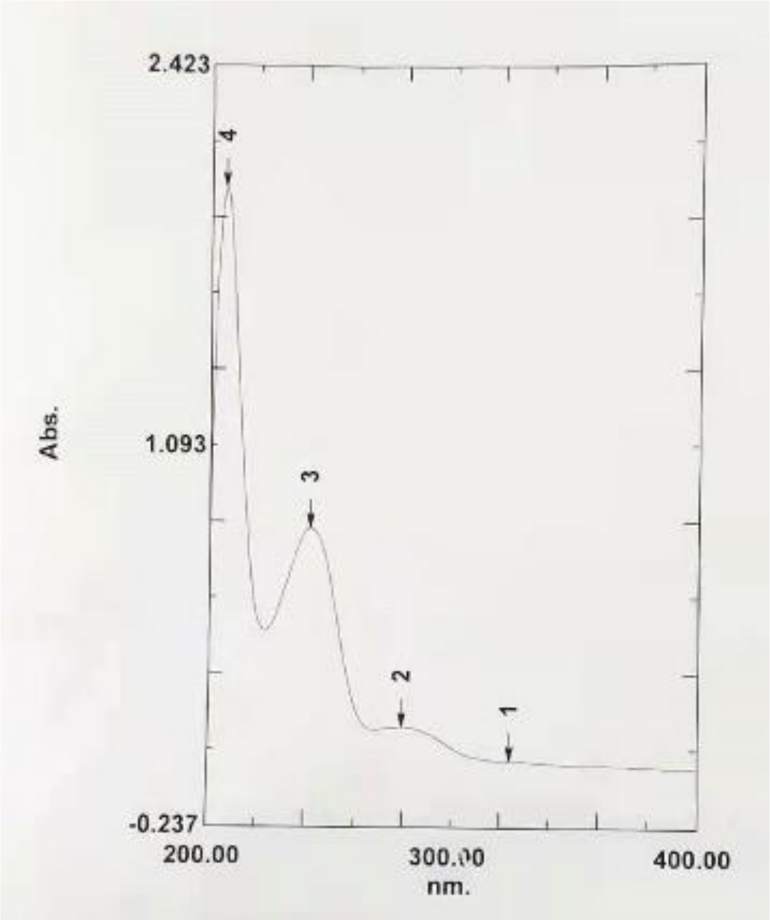

Figure S8. UV spectrum of compound **1**

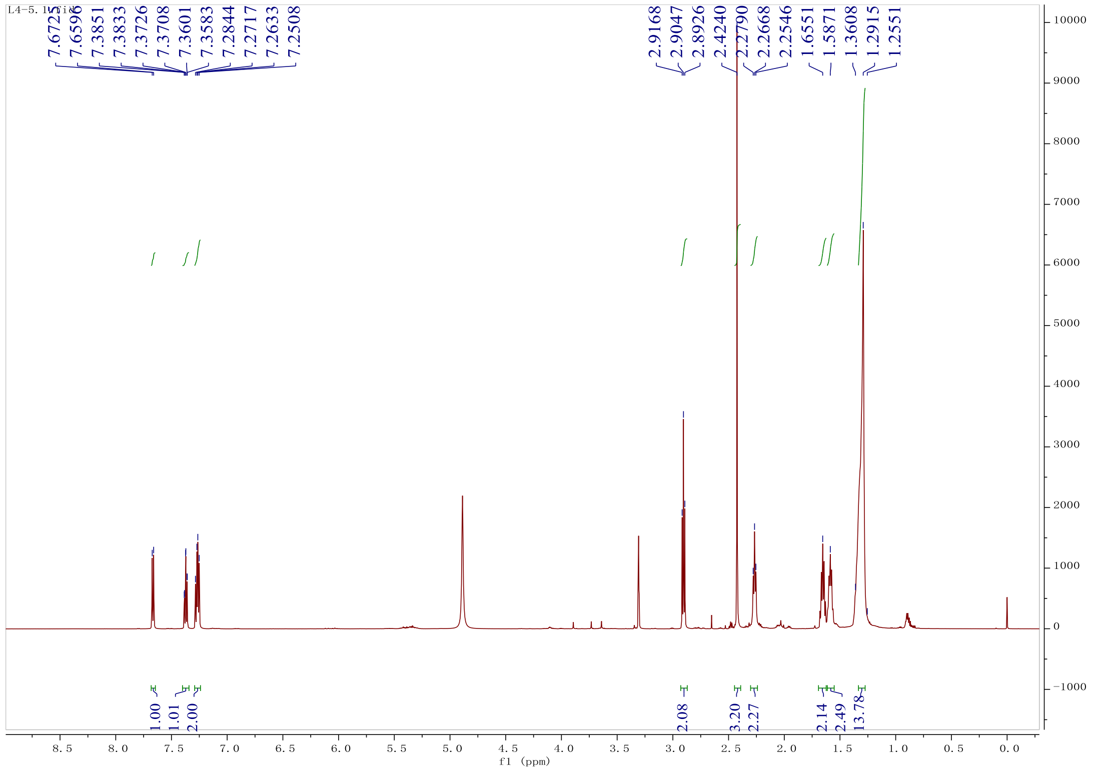

Figure S9. ^{1}H NMR spectrum of compound **2** (CD_3OD, 600 MHz)

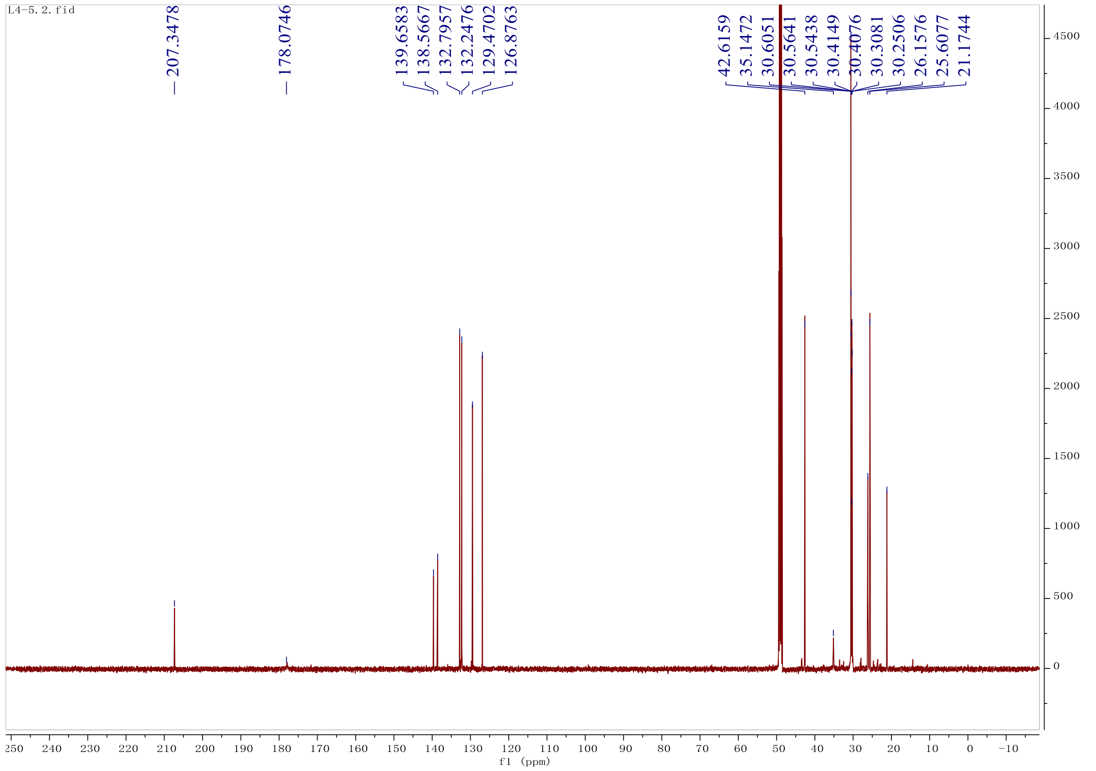

Figure S10. ^{13}C NMR spectrum of compound **2** (CD_3OD, 150 MHz)

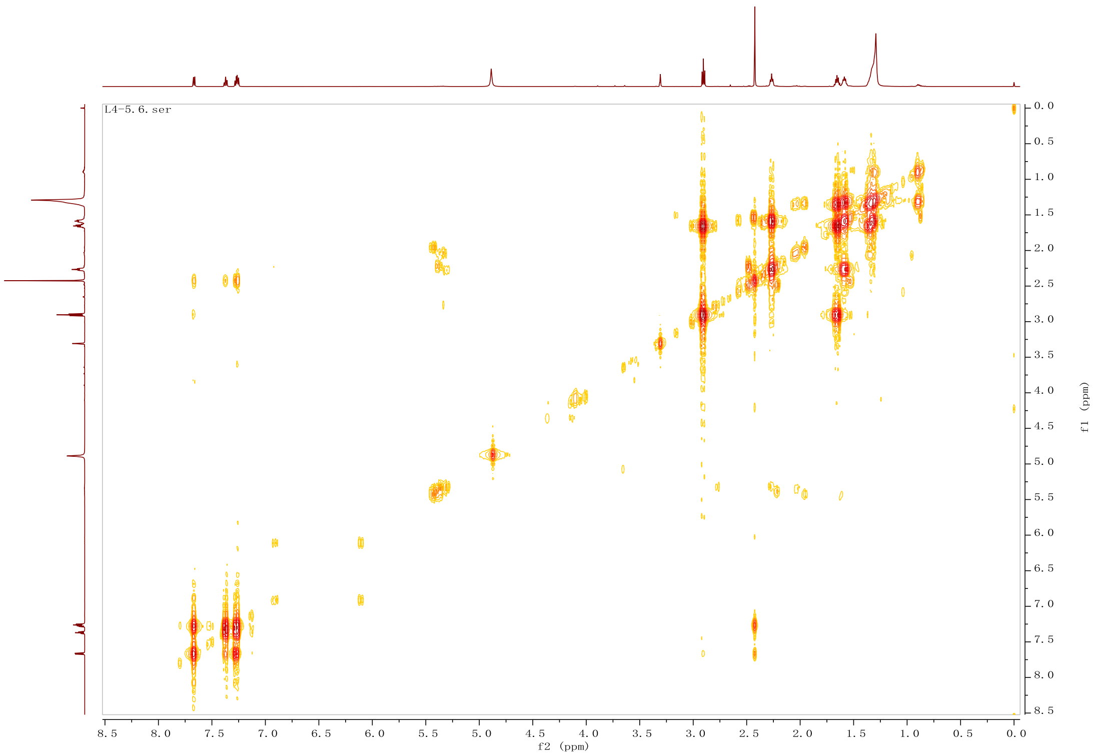

Figure S11. ^{1}H-^{1}HCOSY spectrum of compound **2** (CD_3OD)

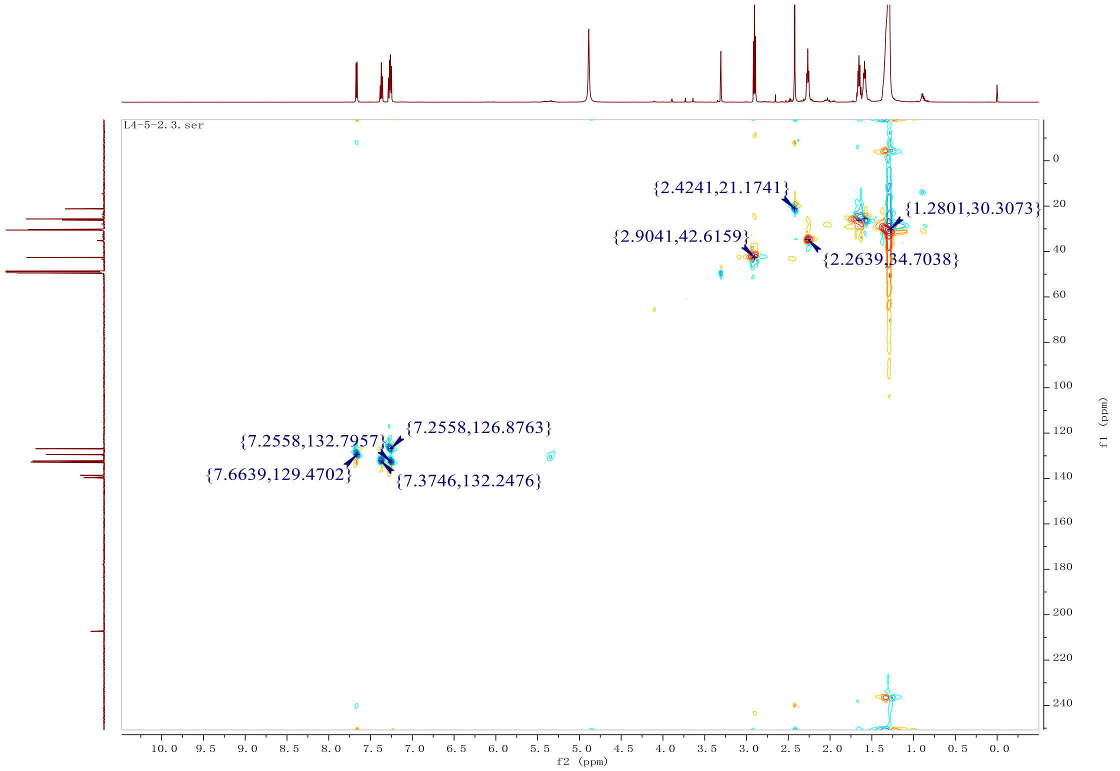

Figure S12. HSQC spectrum of compound **2** (CD_3OD)

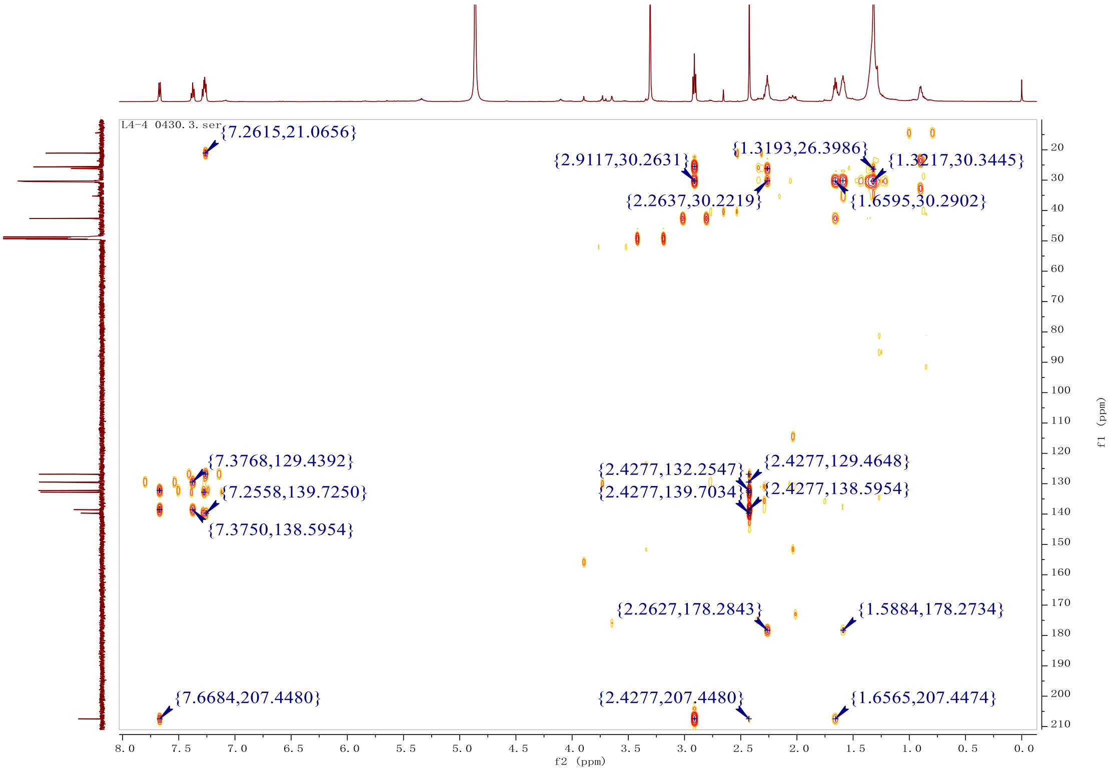

Figure S13. HMBC spectrum of compound **2** (CD_3OD)

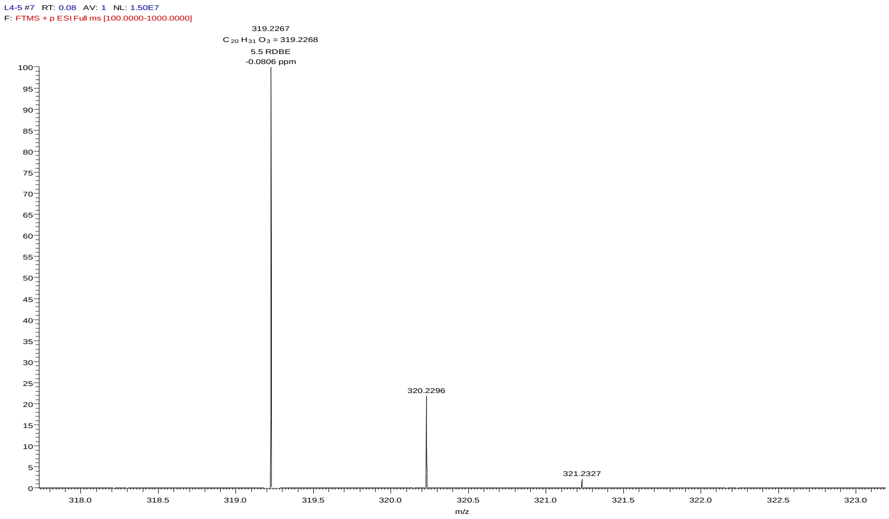

Figure S14. HRESIMS spectrum of compound **2**

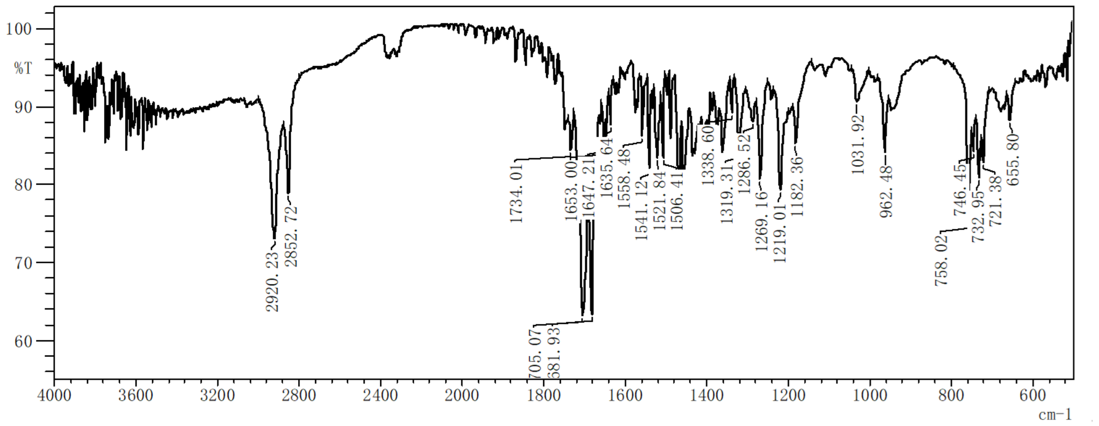

Figure S15. IR spectrum of compound **2**

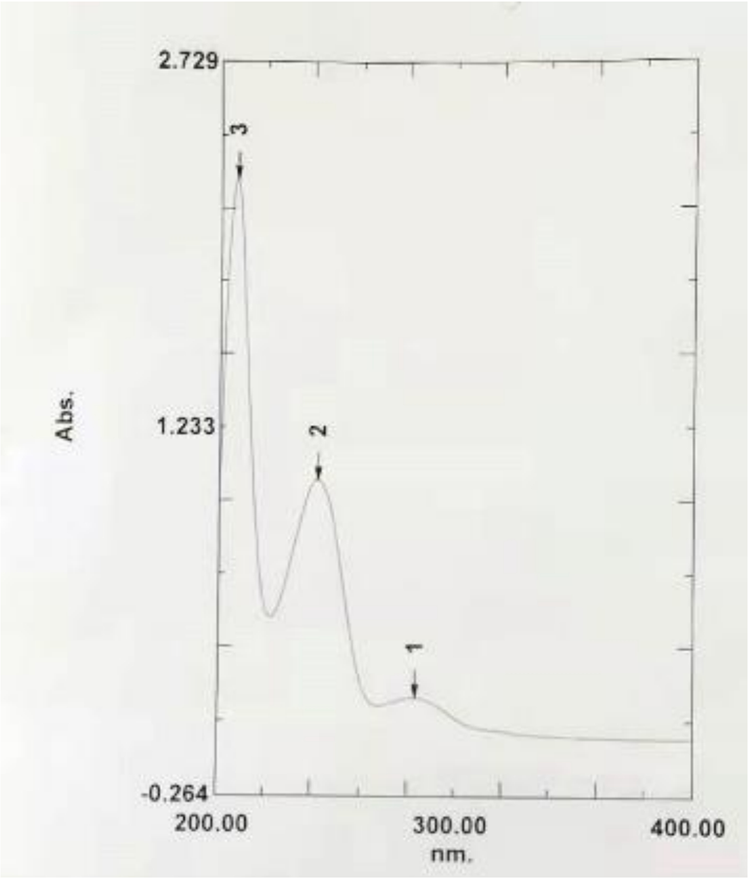

Figure S16. UV spectrum of compound **2**

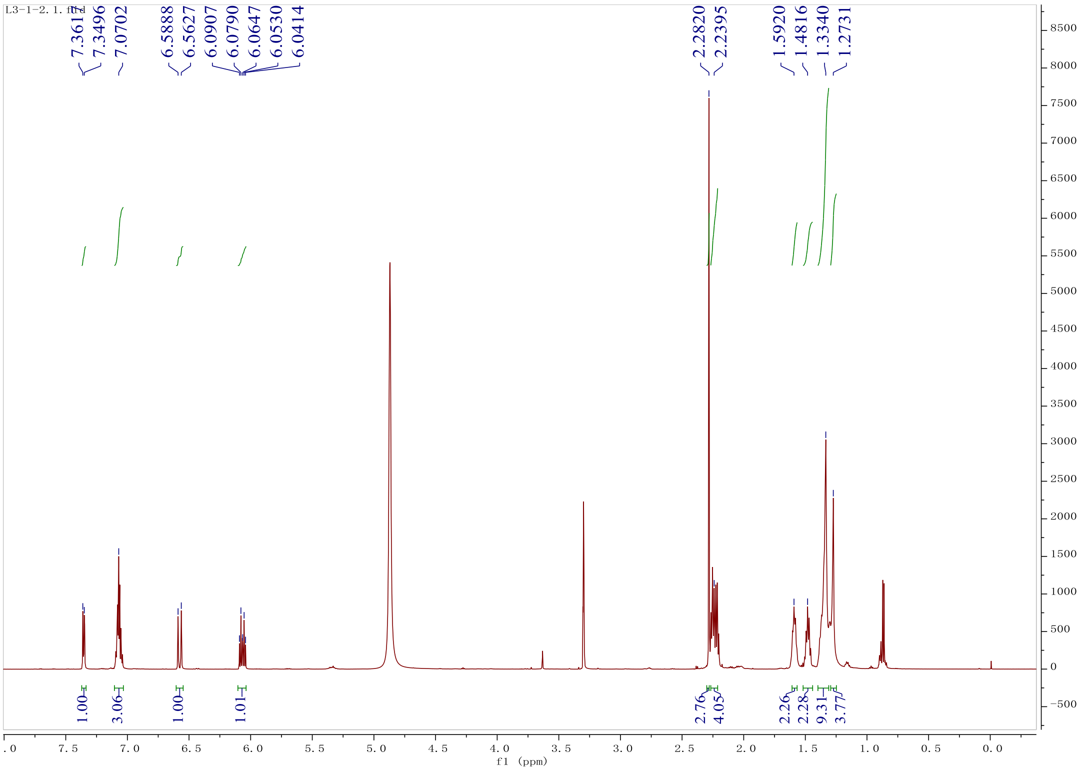

Figure S17. ^{1}H NMR spectrum of compound **3** (CD_3OD, 600 MHz)

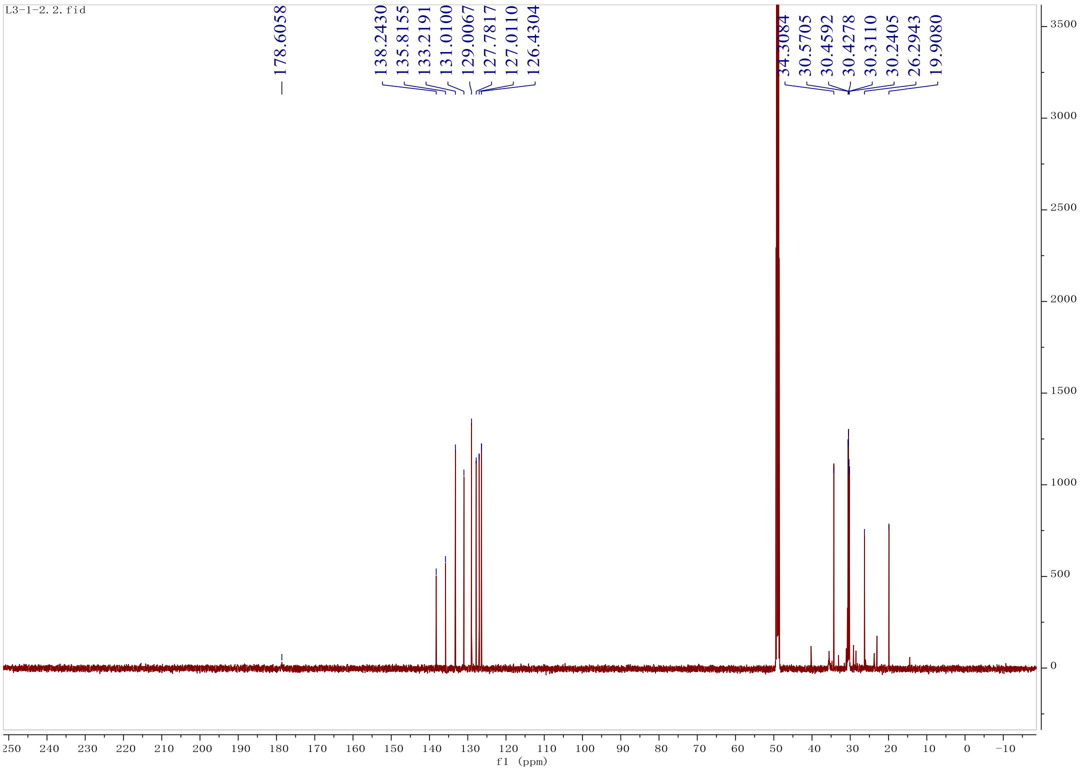

Figure S18. ^{13}C NMR spectrum of compound **3** (CD_3OD, 150 MHz)

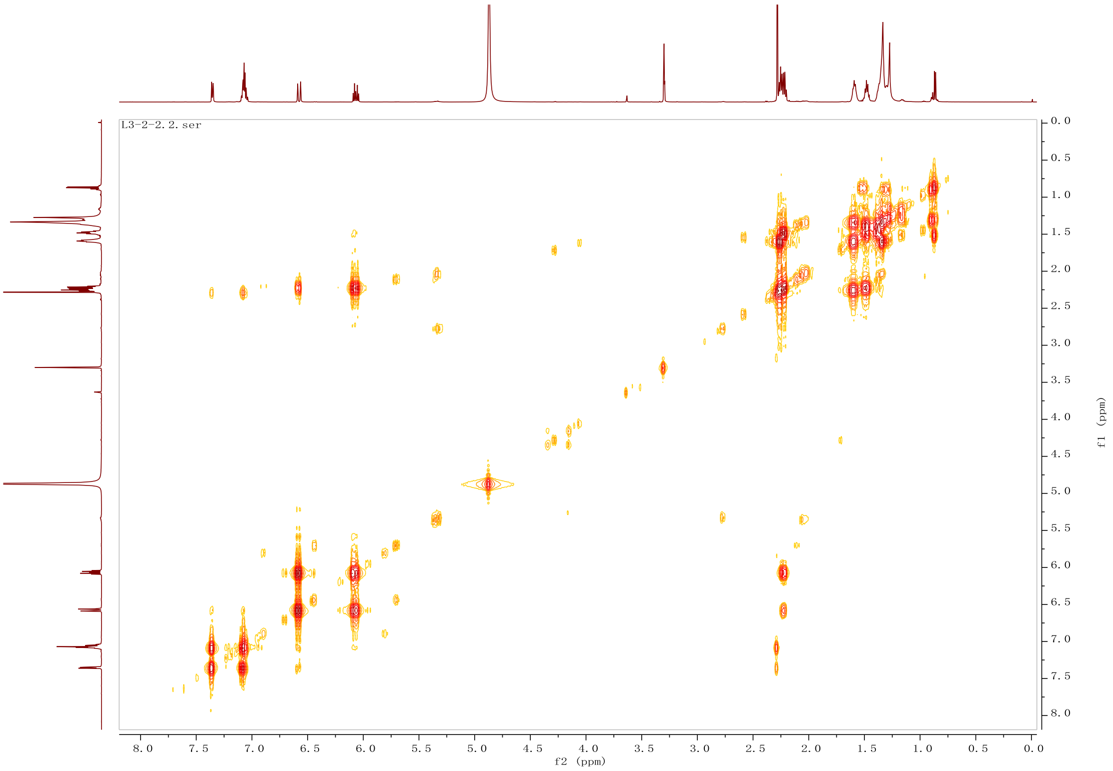

Figure S19. ^{1}H-^{1}HCOSY spectrum of compound **3** (CD_3OD)

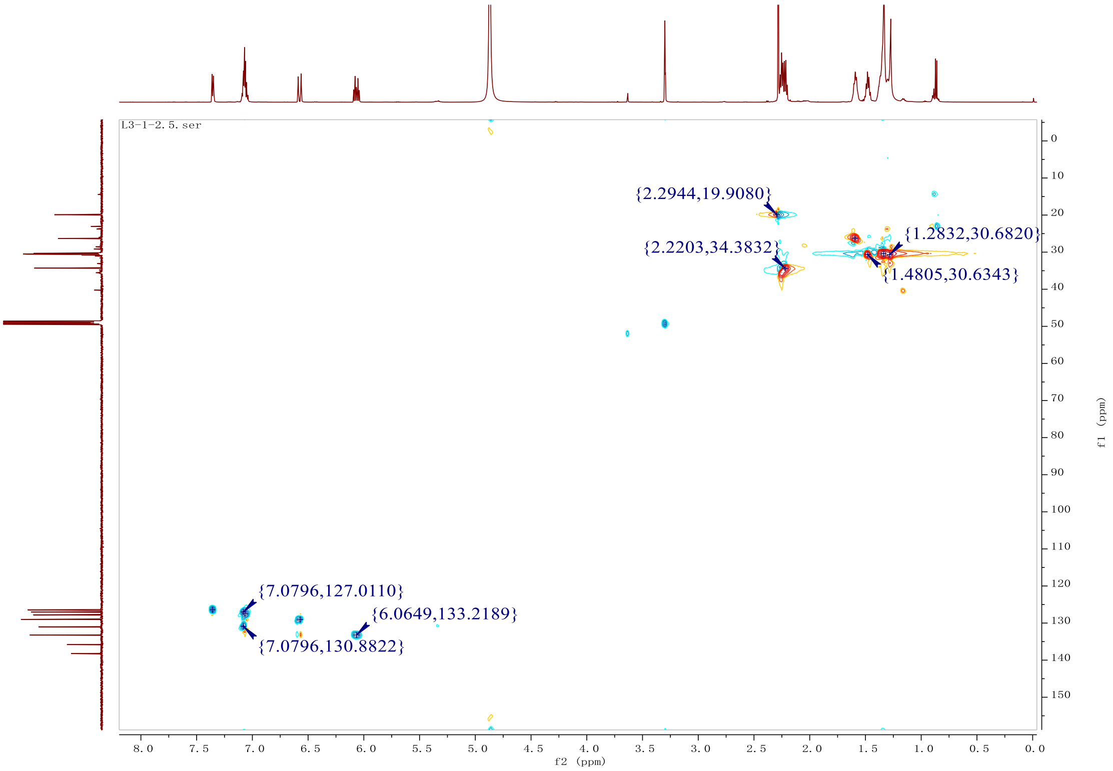

Figure S20. HSQC spectrum of compound **3** (CD_3OD)

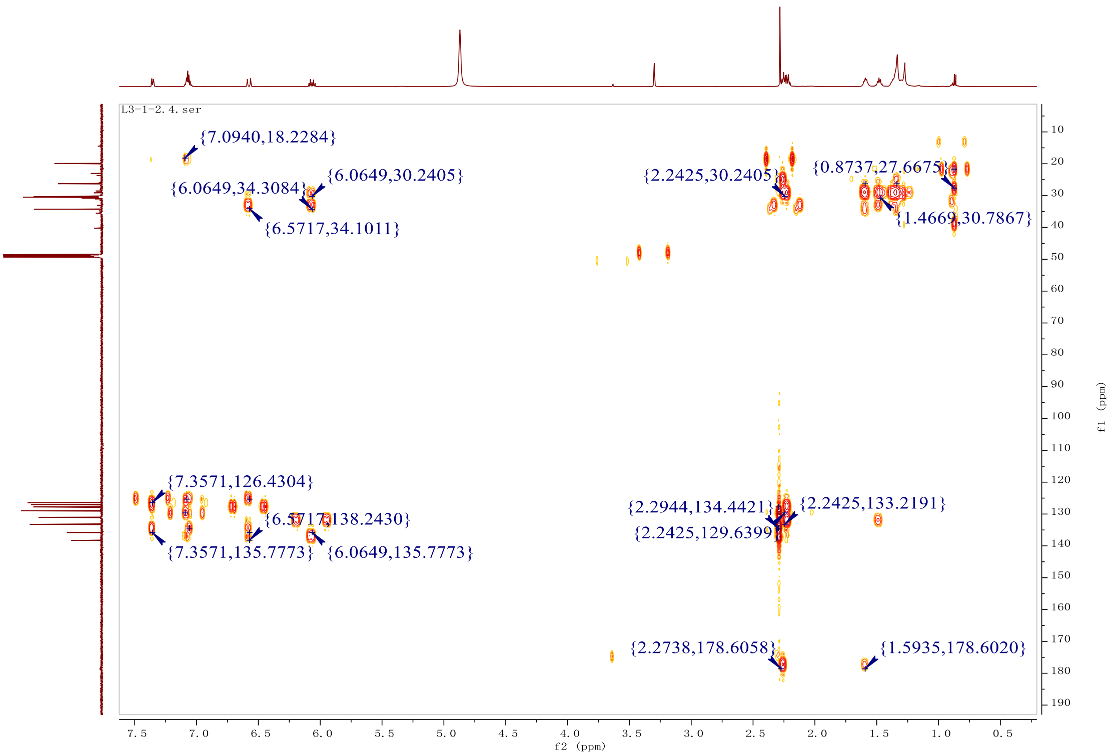

Figure S21. HMBC spectrum of compound **3** (CD_3OD)

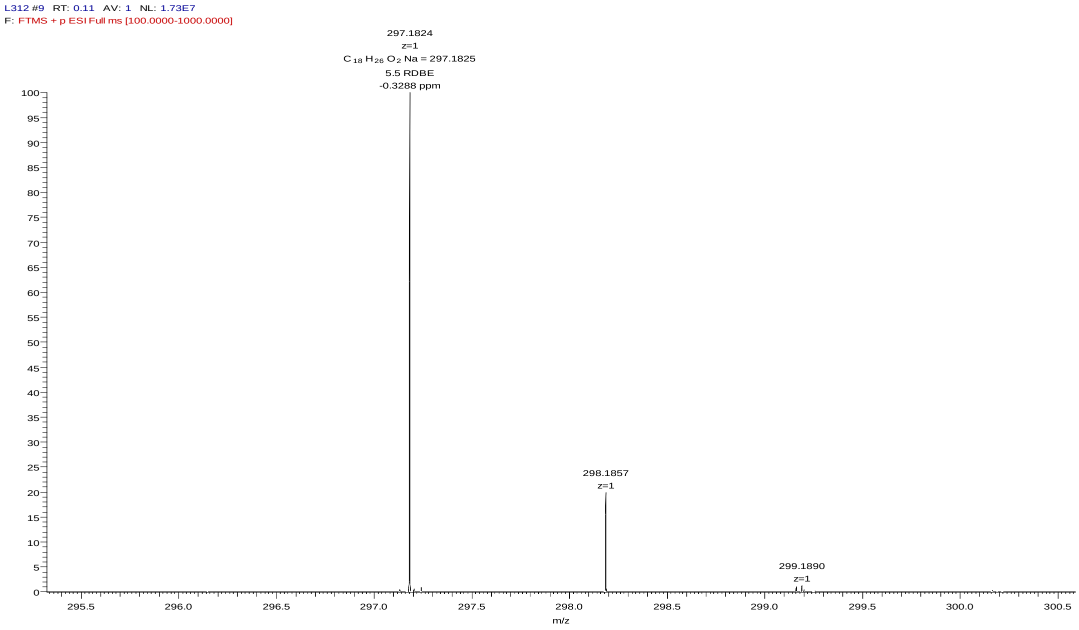

Figure S22. HRESIMS spectrum of compound **3**

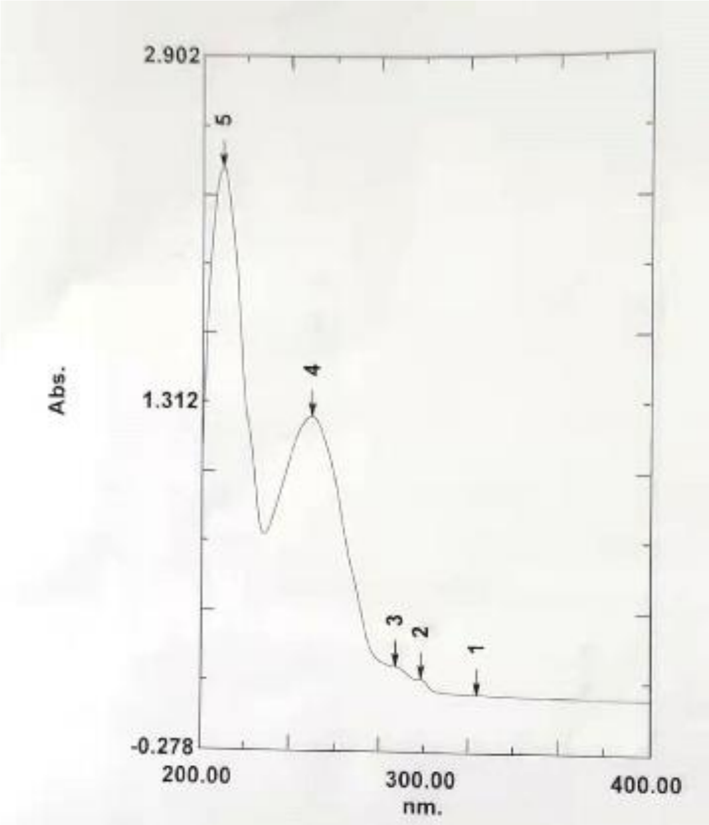

Figure S23. UV spectrum of compound **3**

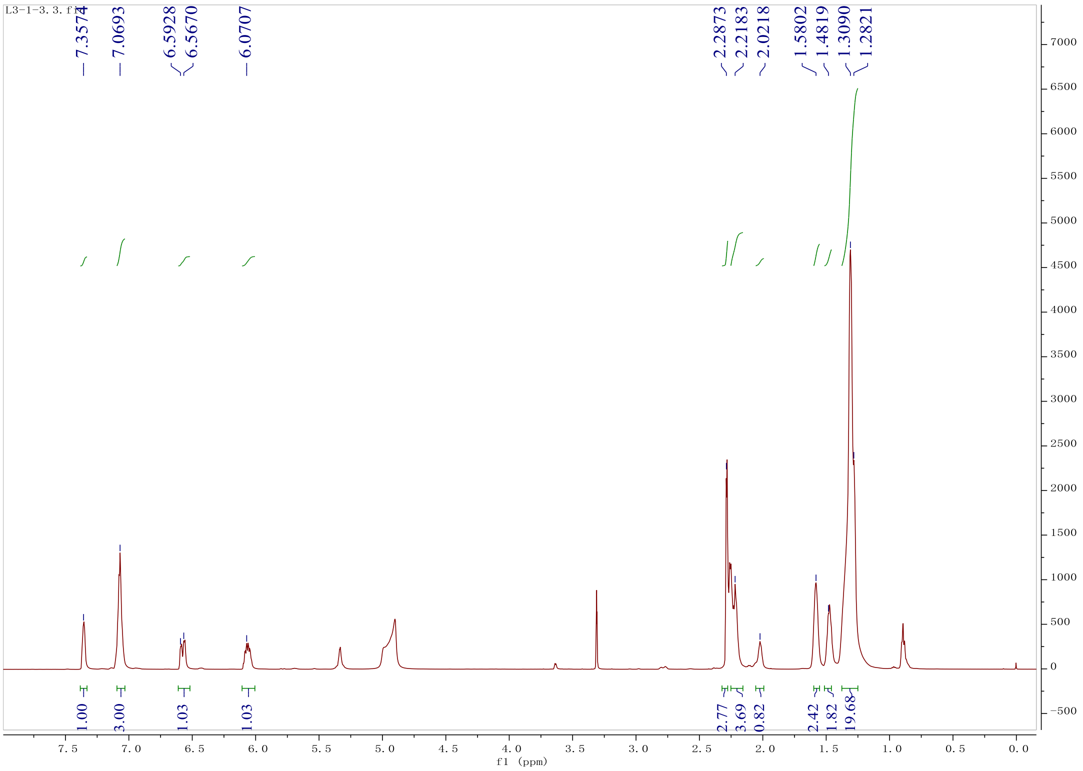

Figure S24. ^{1}H NMR spectrum of compound **4** (CD_3OD, 600 MHz)

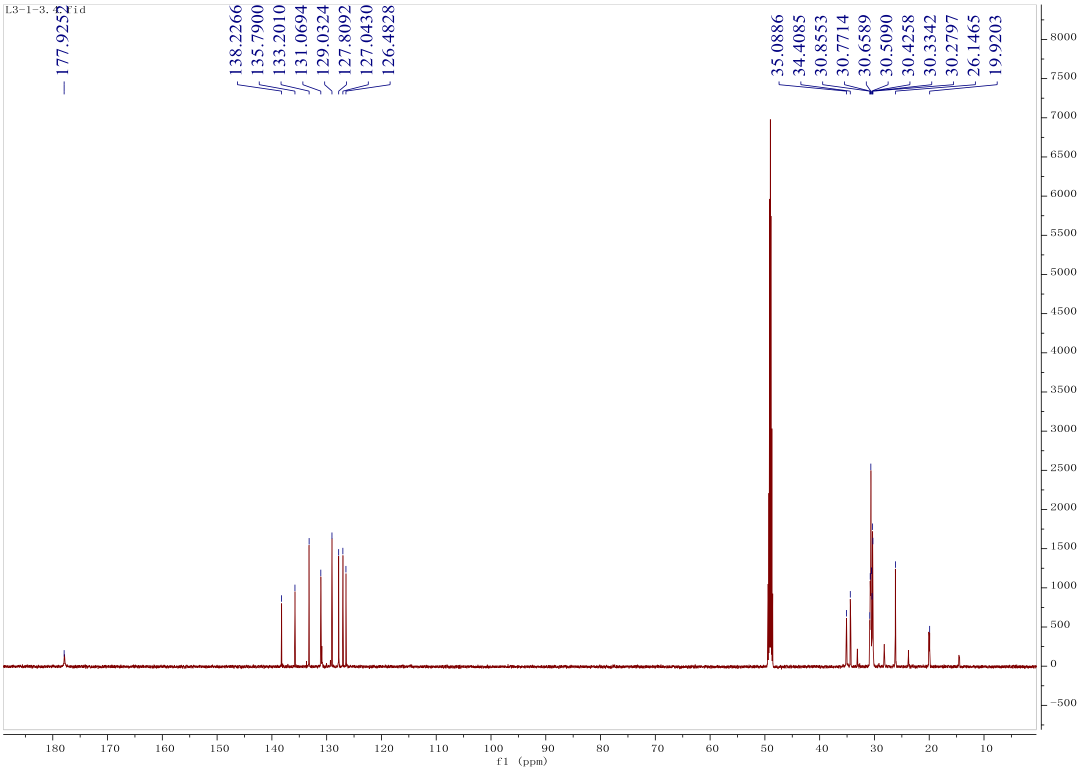

Figure S25. ^{13}C NMR spectrum of compound **4** (CD_3OD, 150 MHz)

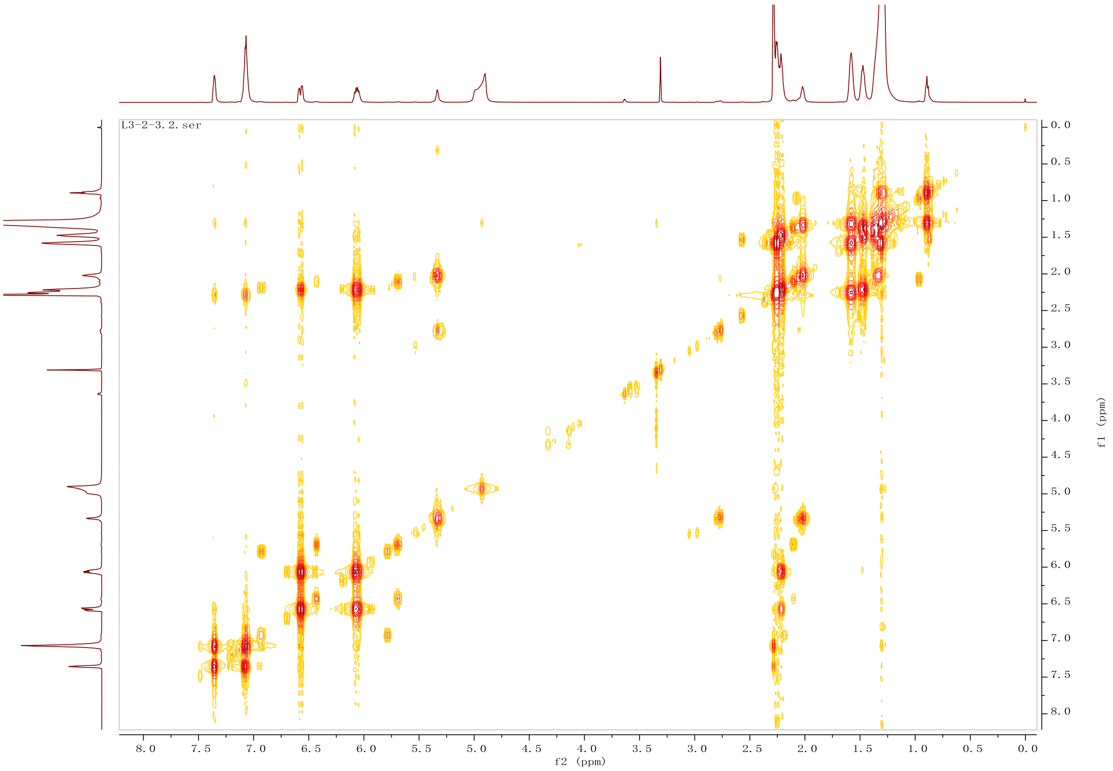

Figure S26. ^{1}H-^{1}HCOSY spectrum of compound **4** (CD_3OD)

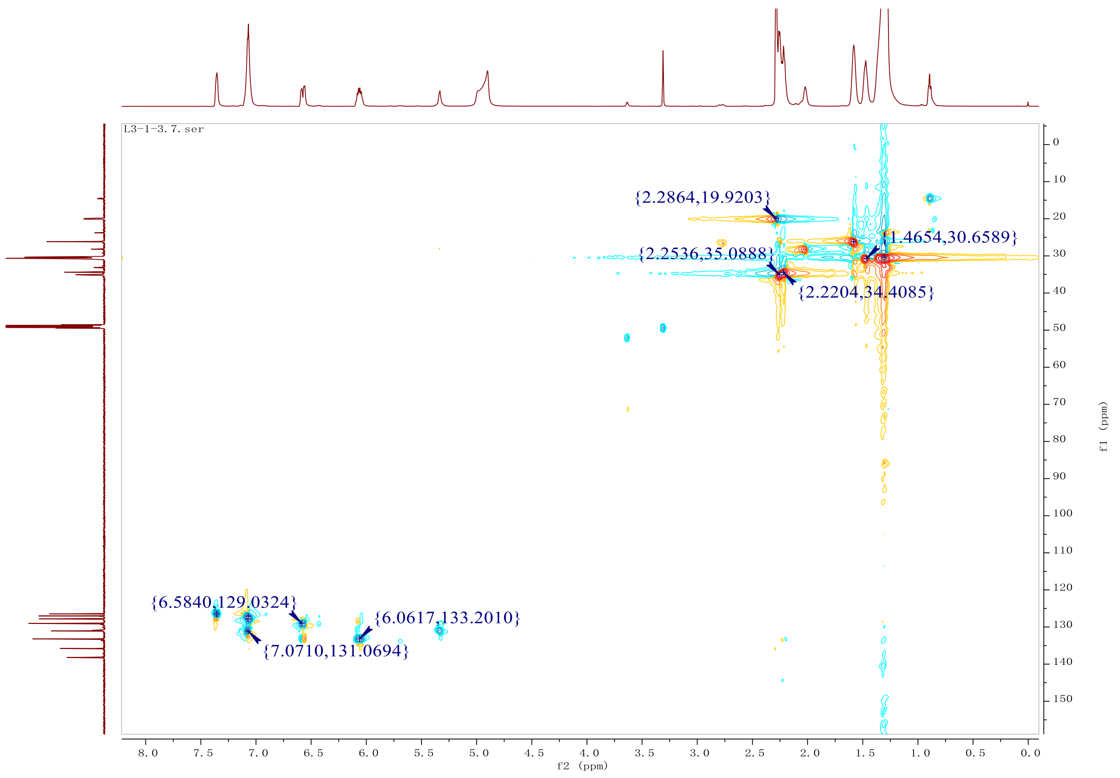

Figure S27. HSQC spectrum of compound **4** (CD_3OD)

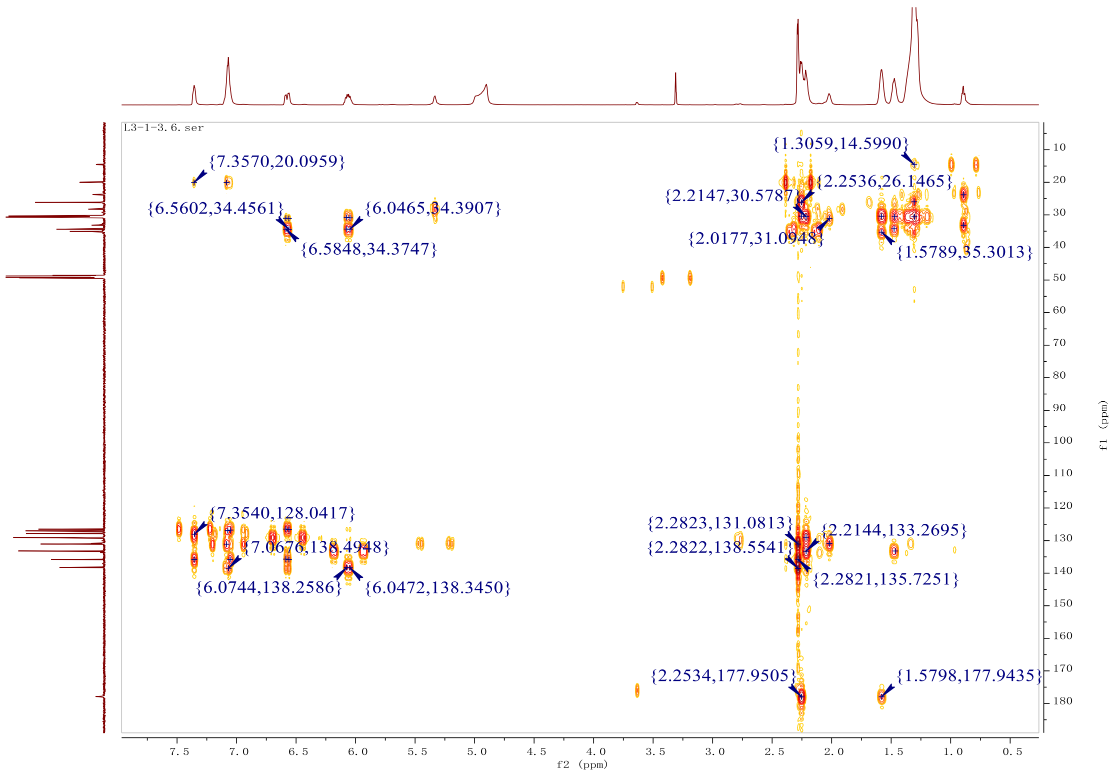

Figure S28. HMBC spectrum of compound **4** (CD_3OD)

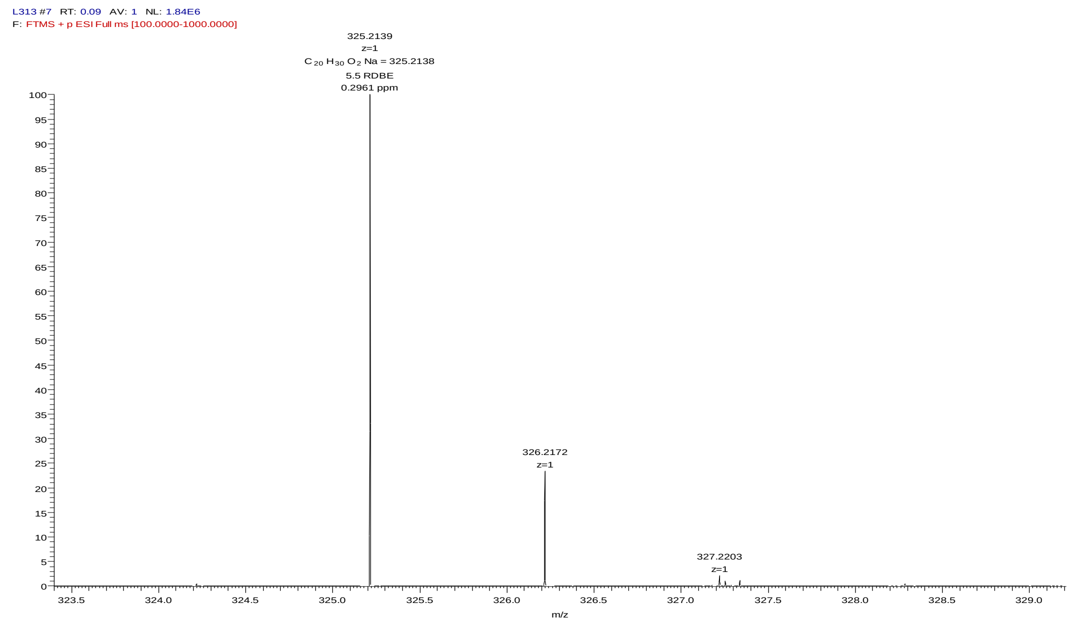

Figure S29. HRESIMS spectrum of compound **4**

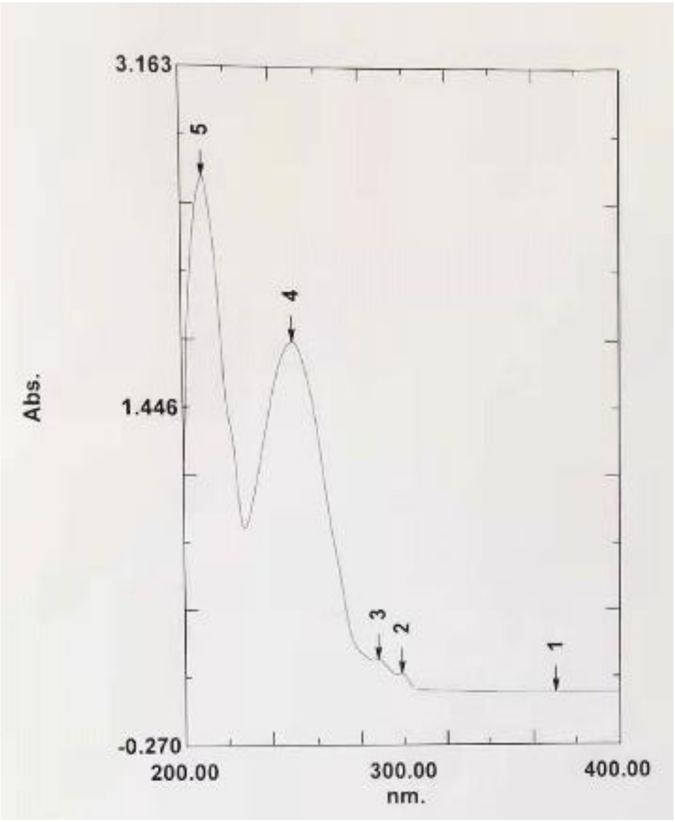

Figure S30. UV spectrum of compound **4**

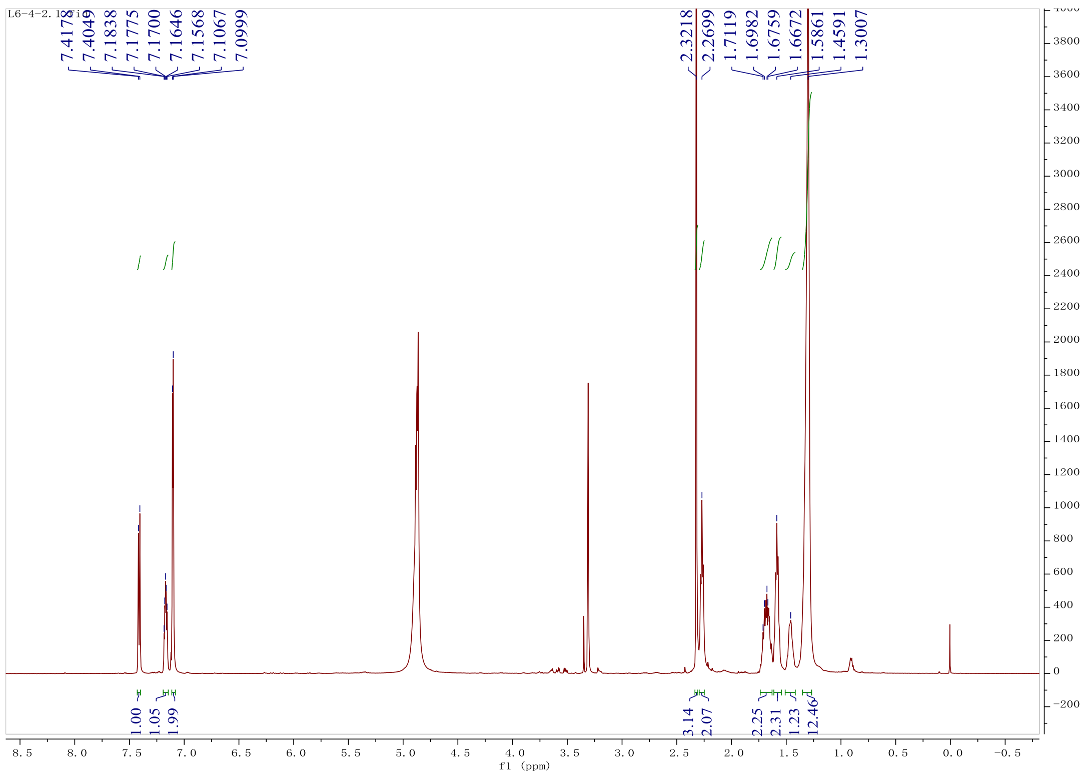

Figure S31. ^{1}H NMR spectrum of compound **5** (CD_3OD, 600 MHz)

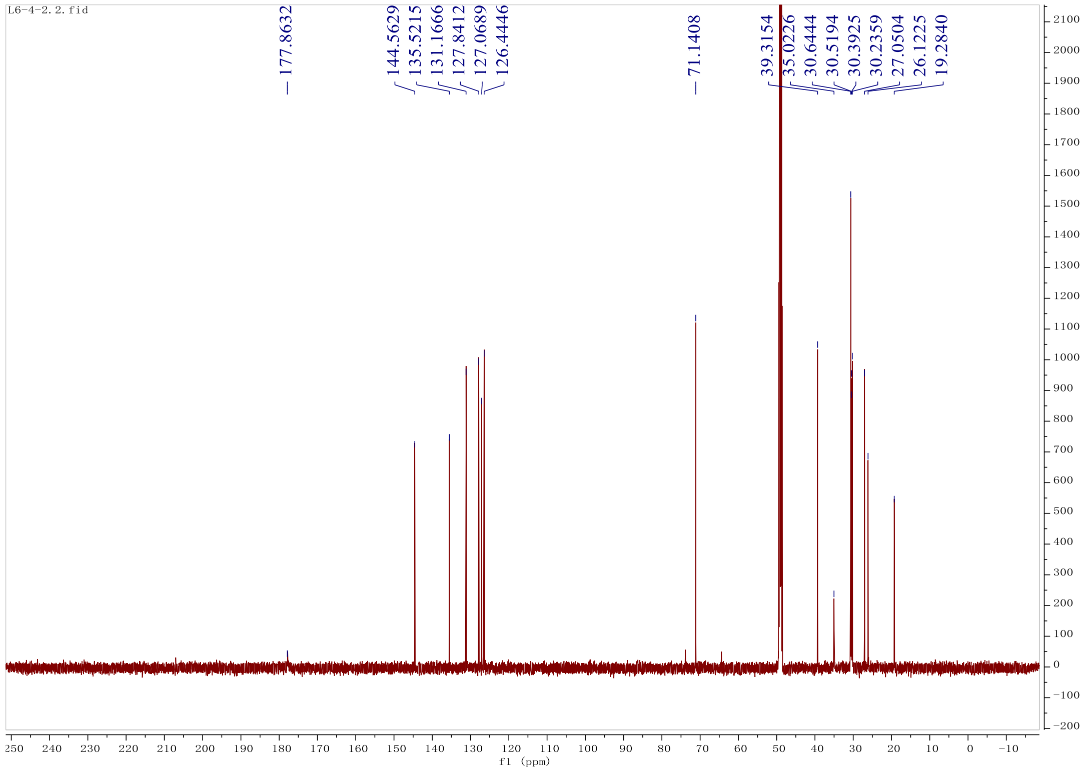

Figure S32. ^{13}C NMR spectrum of compound **5** (CD_3OD, 150 MHz)

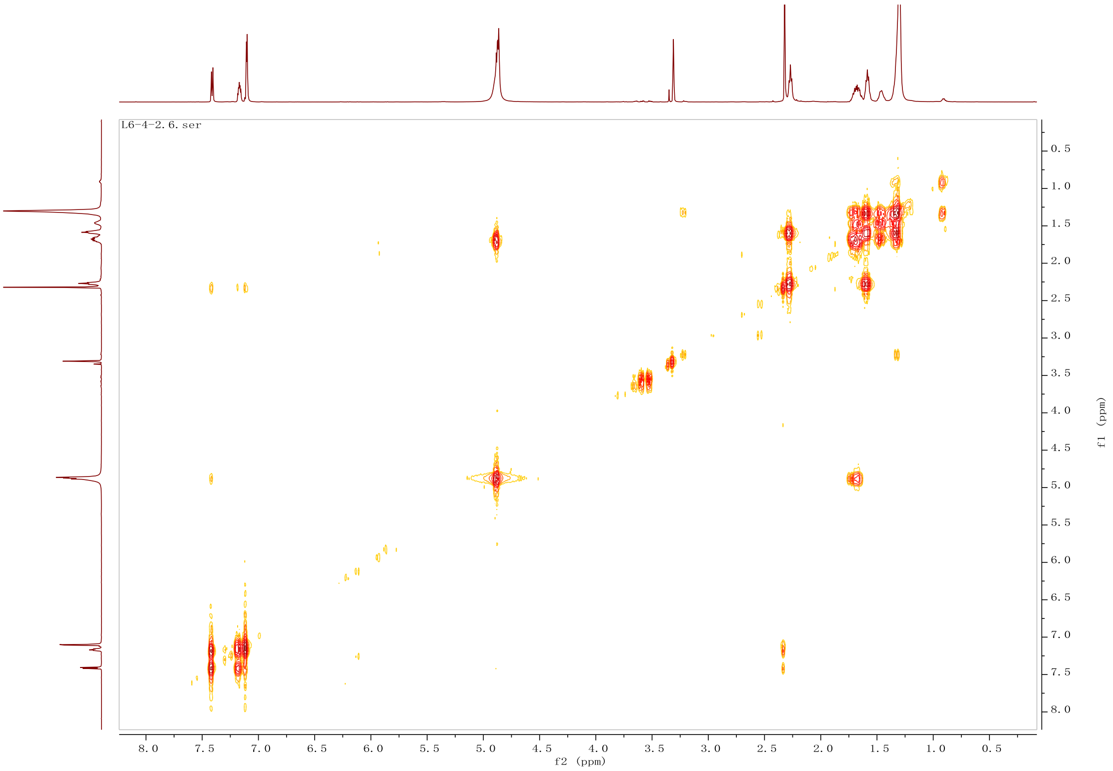

Figure S33. ^1H-^1HCOSY spectrum of compound **5** (CD_3OD)

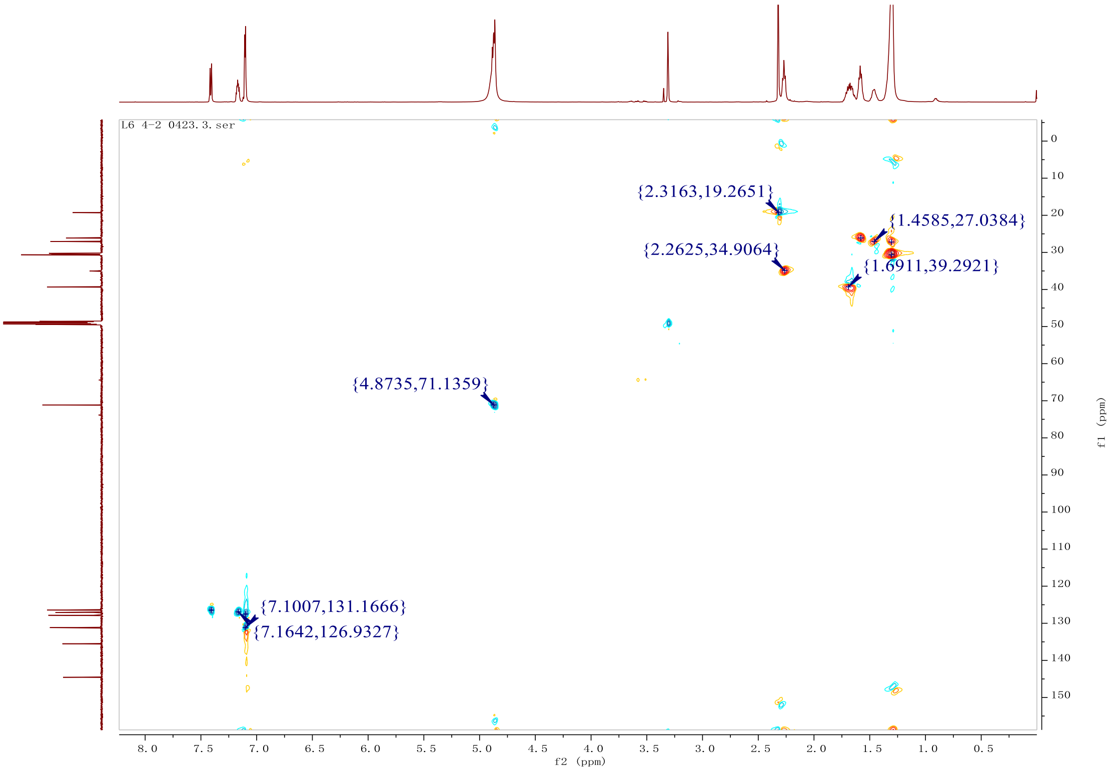

Figure S34. HSQC spectrum of compound **5** (CD_3OD)

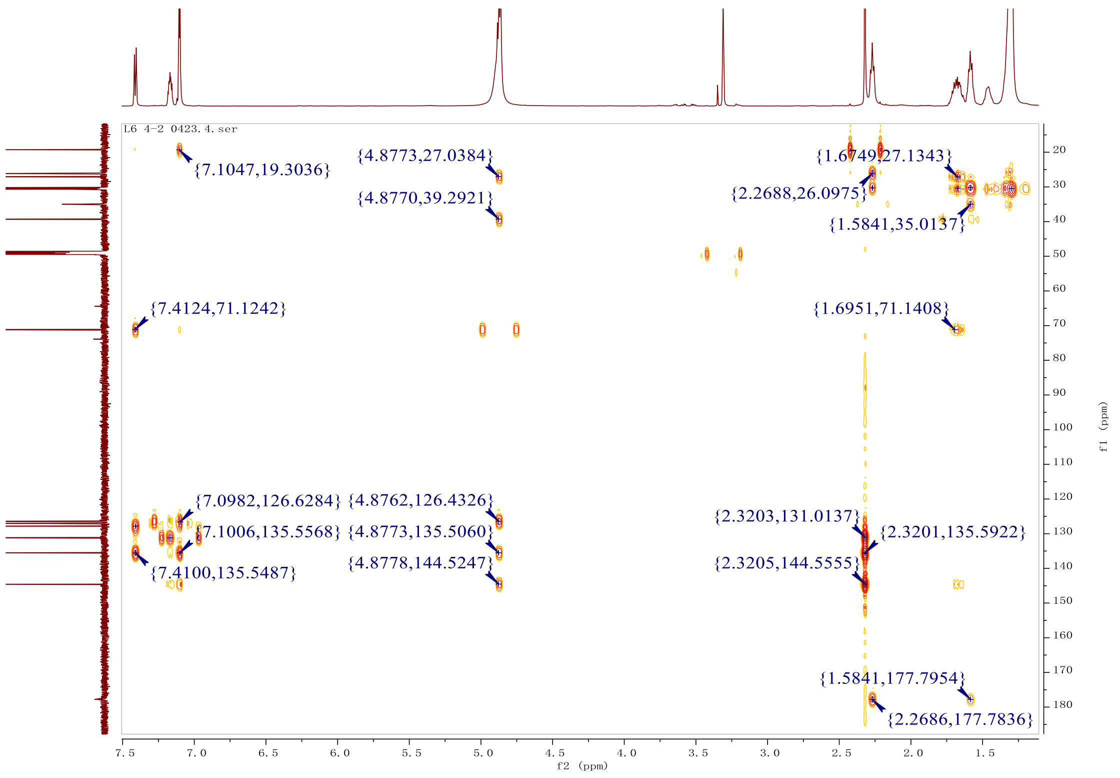

Figure S35. HMBC spectrum of compound **5** (CD_3OD)

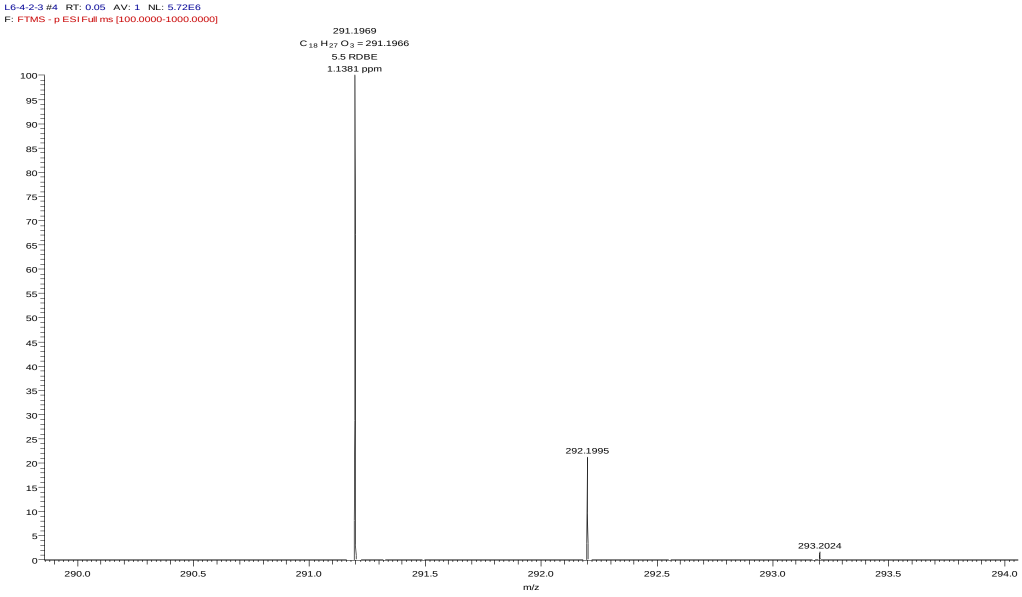

Figure S36. HRESIMS spectrum of compound **5**

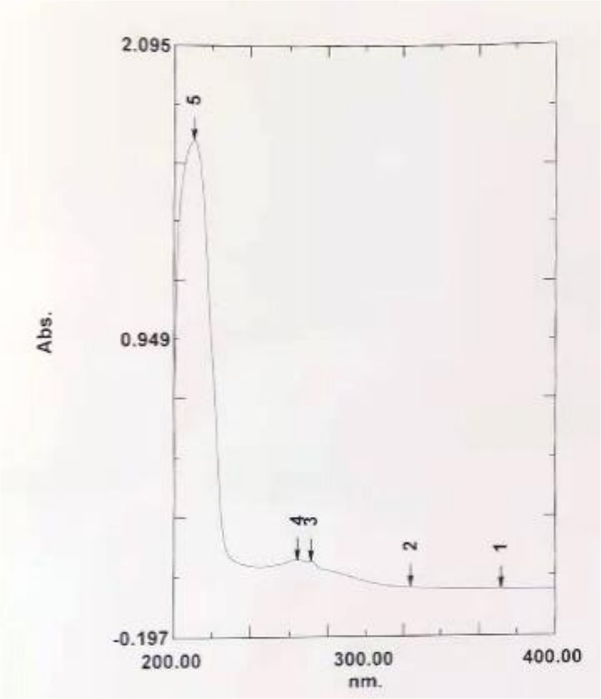

Figure S37. UV spectrum of compound **5**

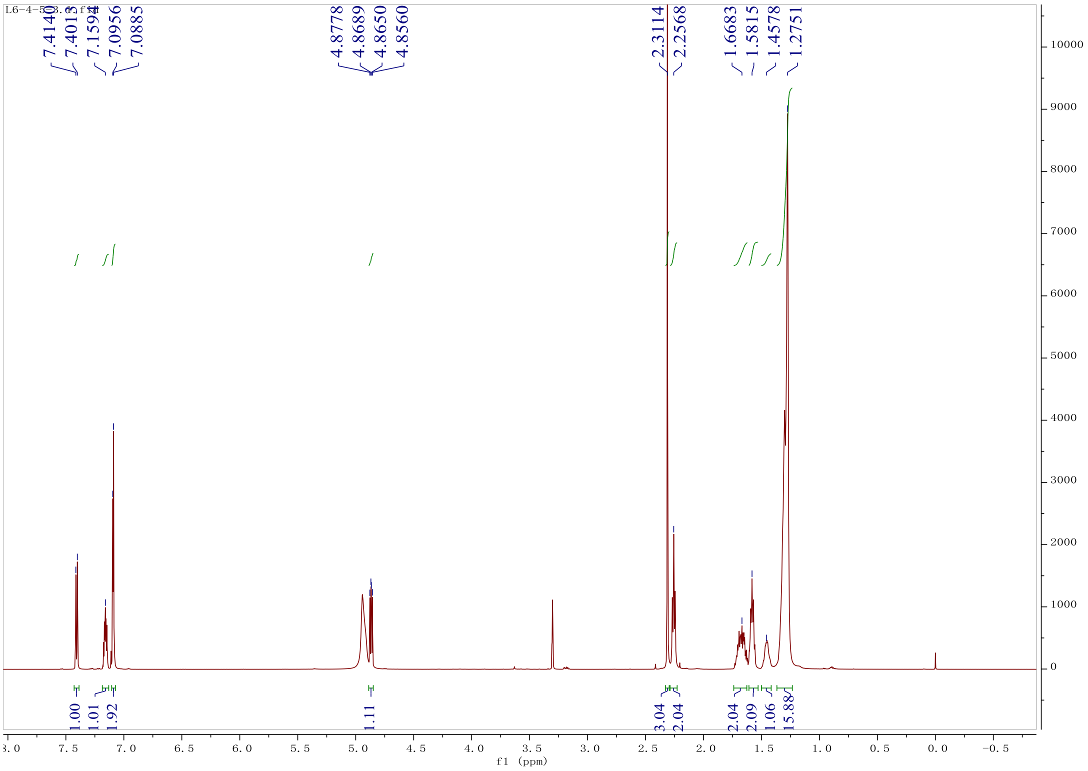

Figure S38. ^{1}H NMR spectrum of compound **6** (CD_3OD, 600 MHz)

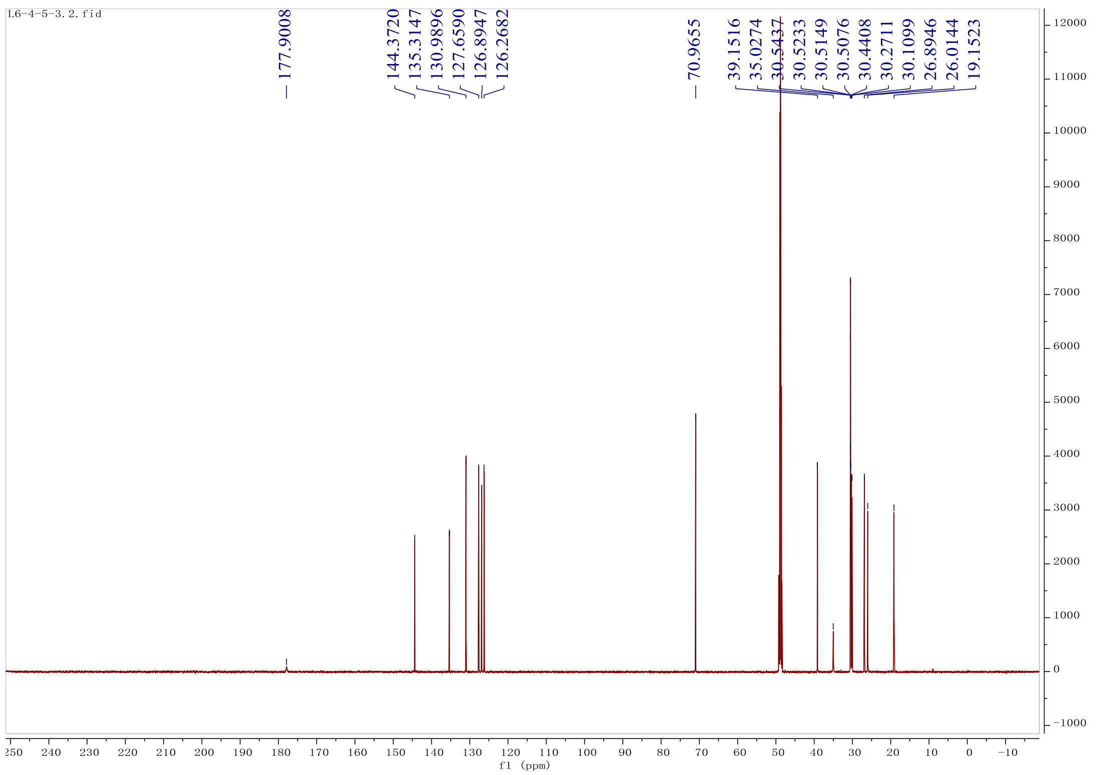

Figure S39. ^{13}C NMR spectrum of compound **6** (CD_3OD, 150 MHz)

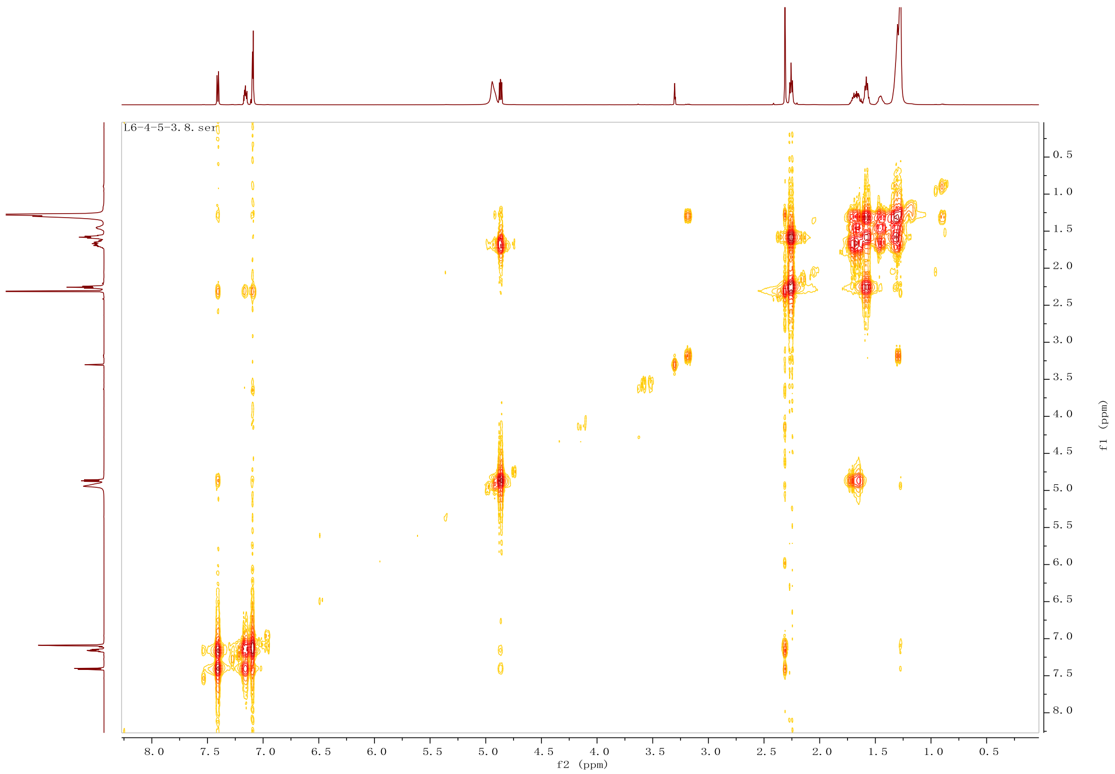

Figure S40. ^{1}H-^{1}HCOSY spectrum of compound **6** (CD_3OD)

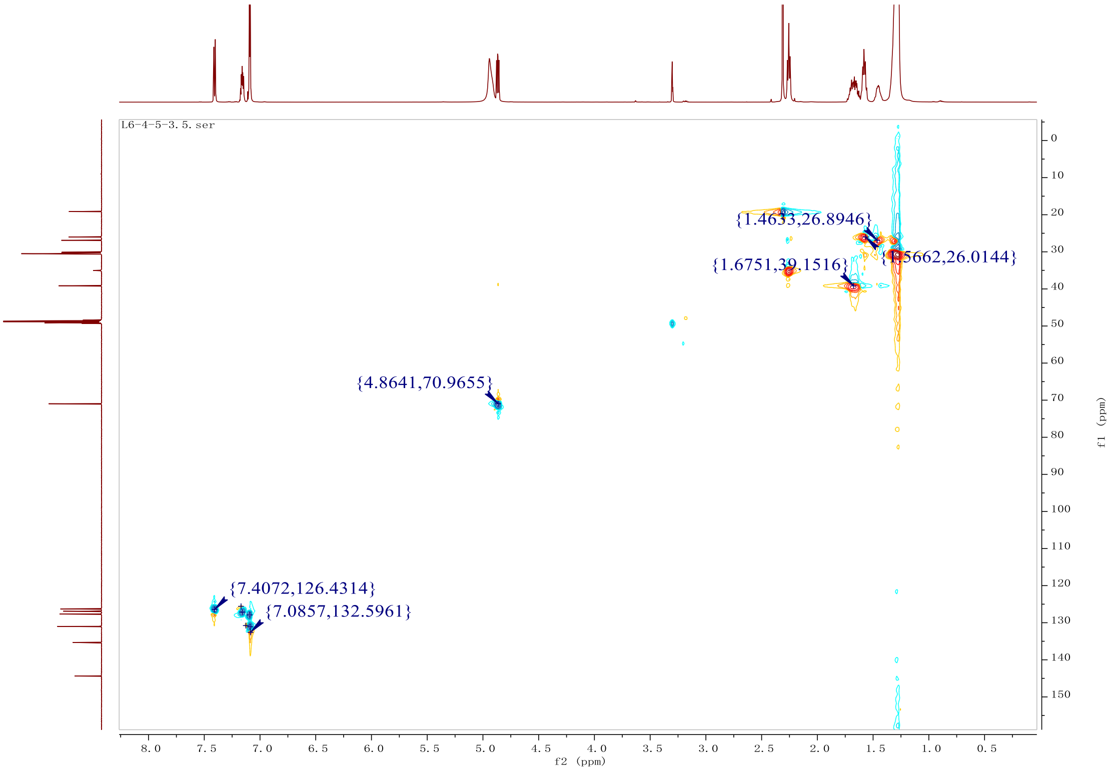

Figure S41. HSQC spectrum of compound **6** (CD_3OD)

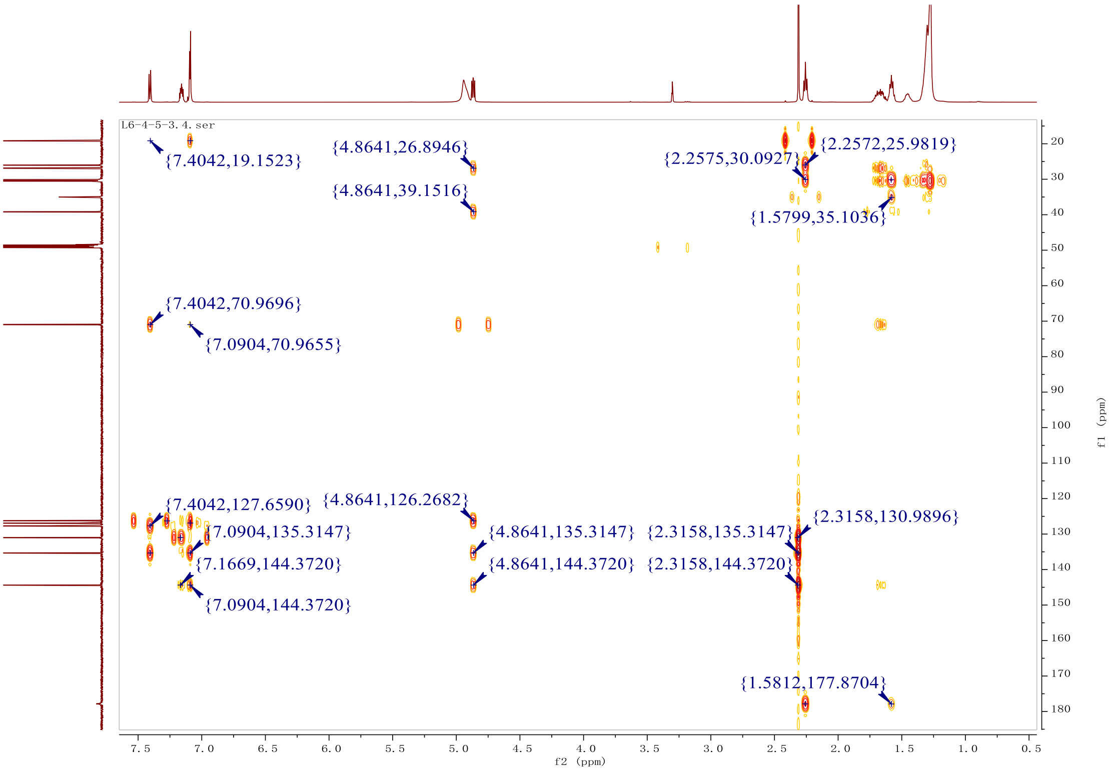

Figure S42. HMBC spectrum of compound **6** (CD_3OD)

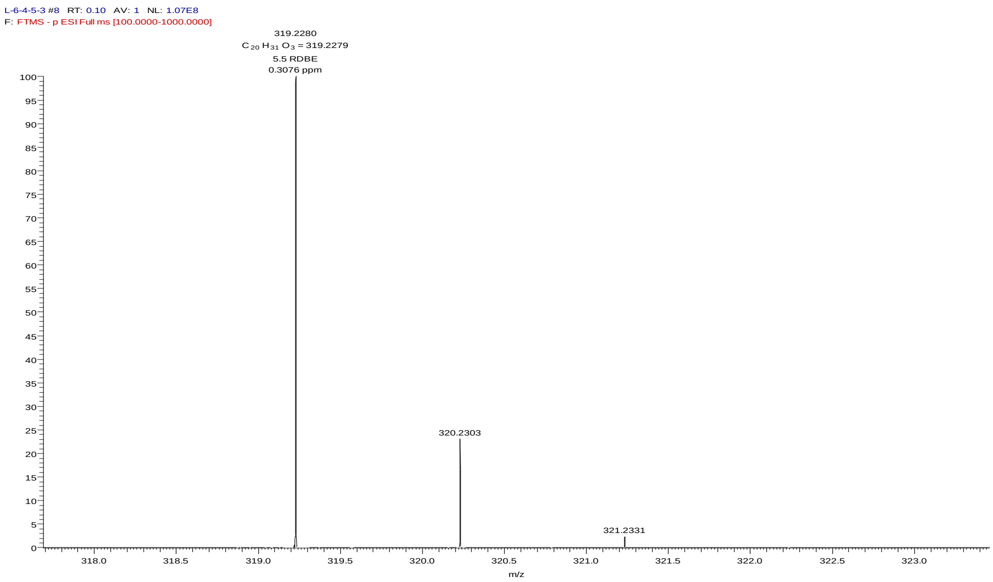

Figure S43. HRESIMS spectrum of compound **6**

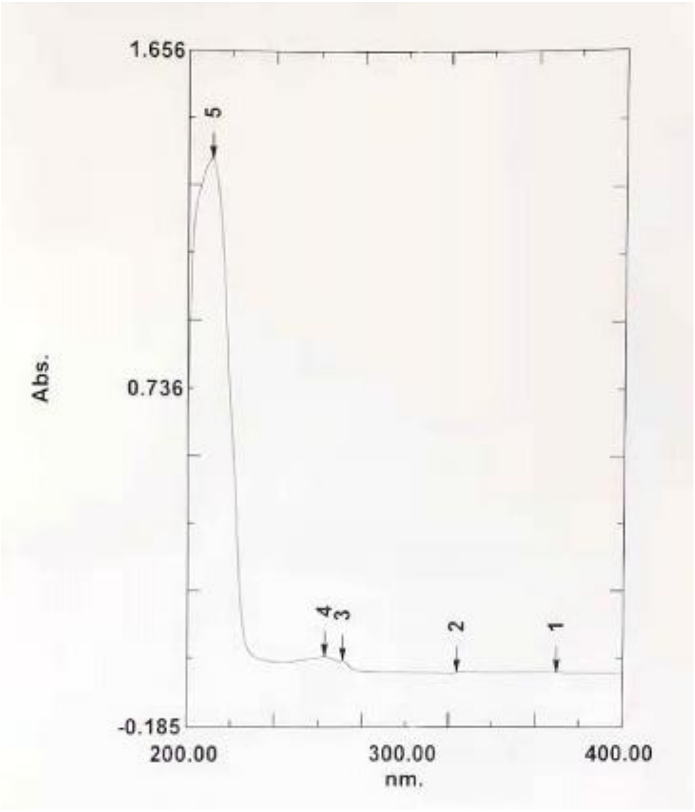

Figure S44. UV spectrum of compound **6**

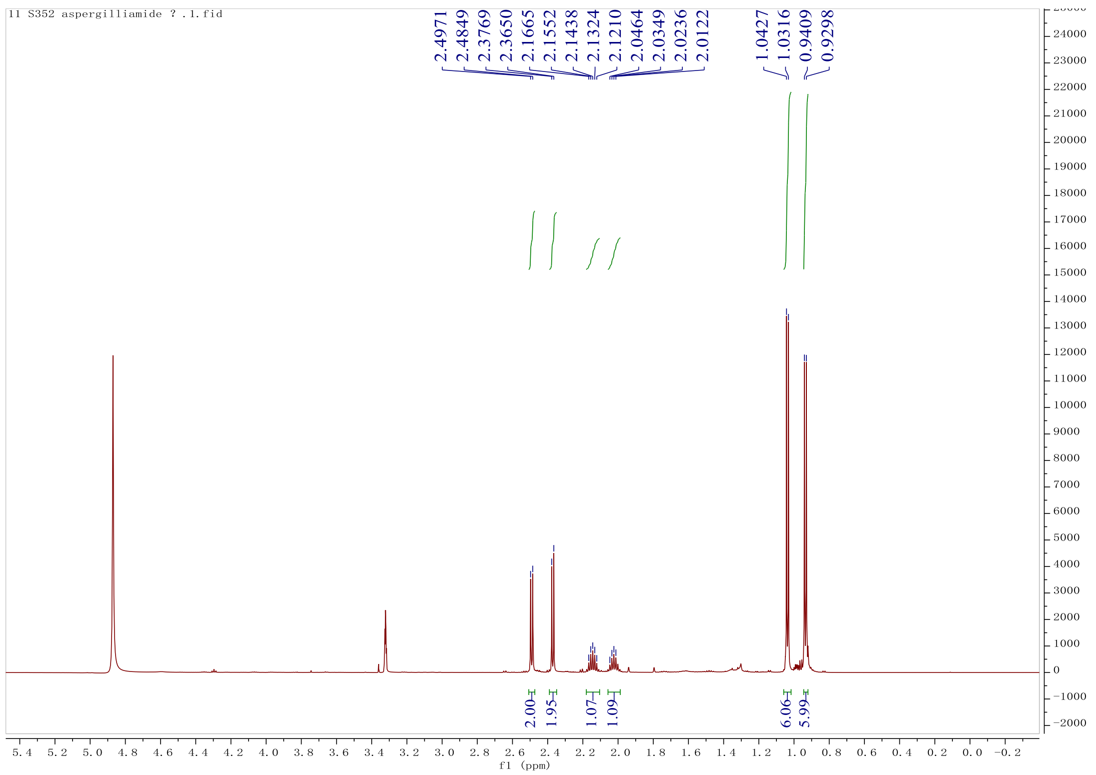

Figure S45. ^{1}H NMR spectrum of compound **7** (CD_3OD, 600 MHz)

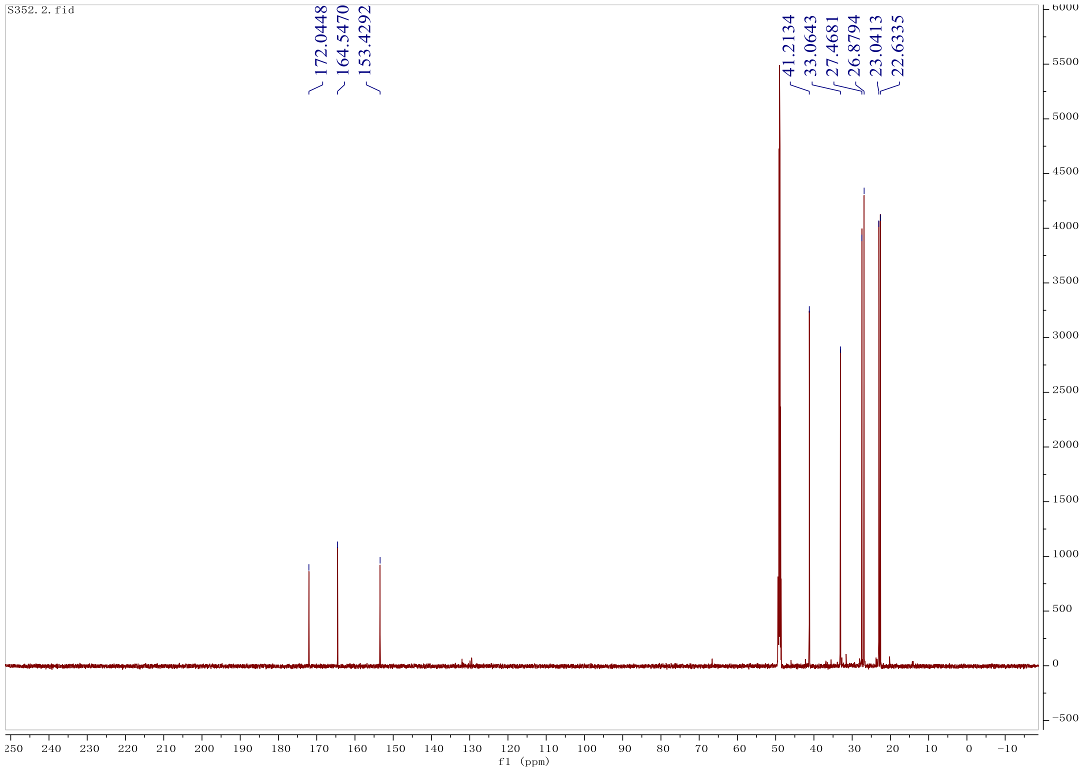

Figure S46. ^{13}C NMR spectrum of compound **7** (CD_3OD, 150 MHz)

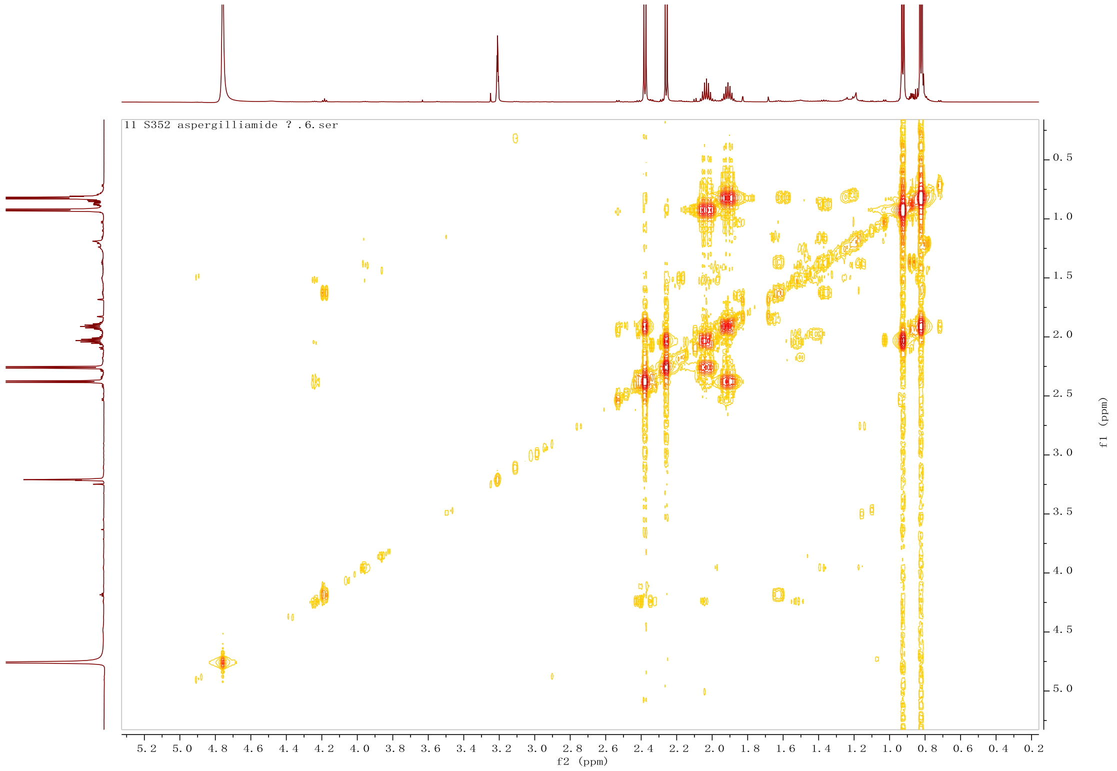

Figure S47. ^{1}H-^{1}HCOSY spectrum of compound **7** (CD_3OD)

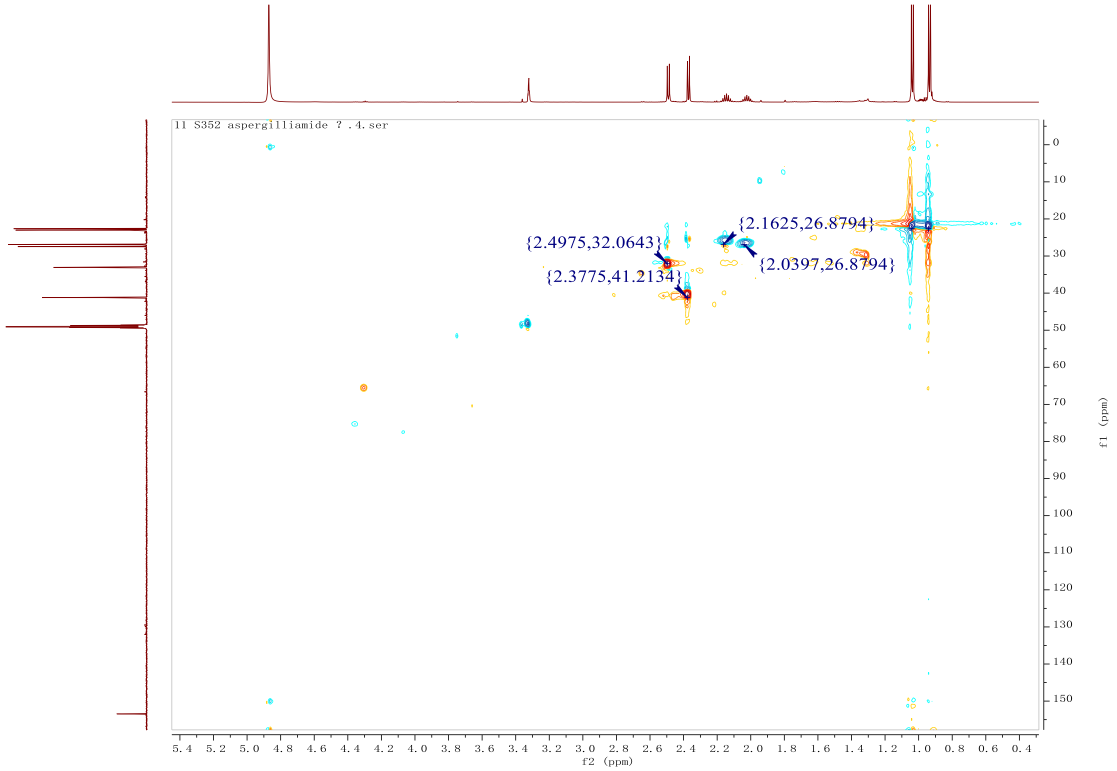

Figure S48. HSQC spectrum of compound **7** (CD_3OD)

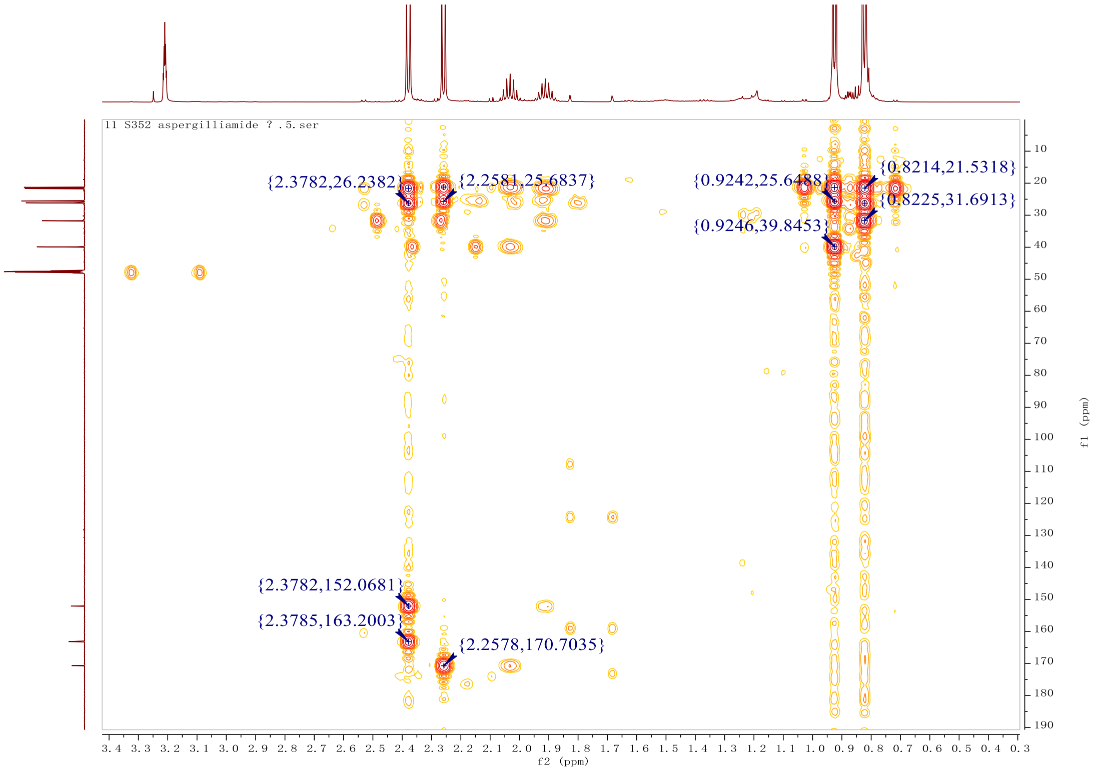

Figure S49. HMBC spectrum of compound **7** (CD_3OD)

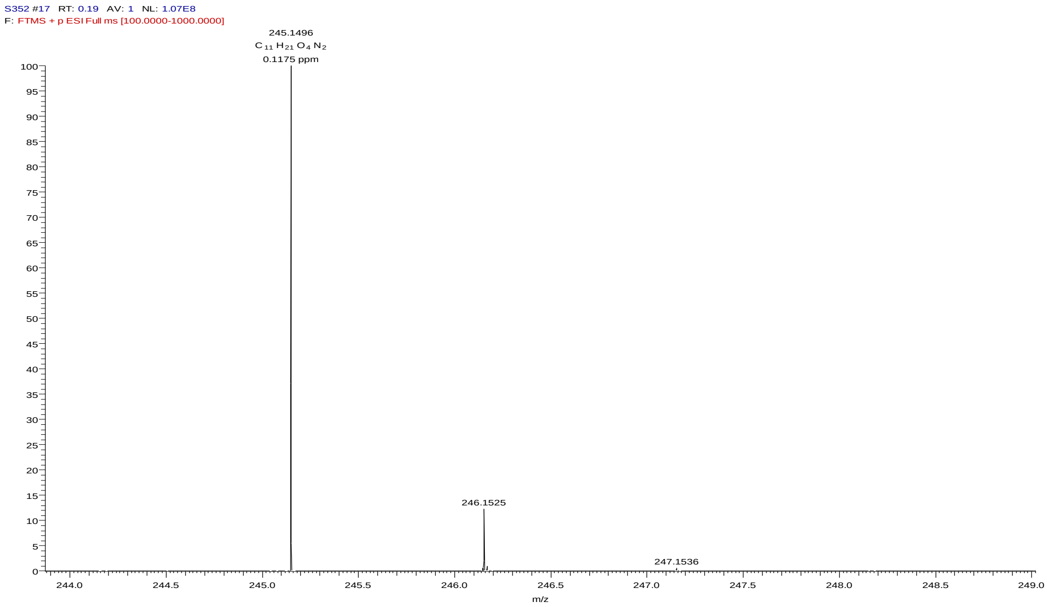

Figure S50. HRESIMS spectrum of compound **7**

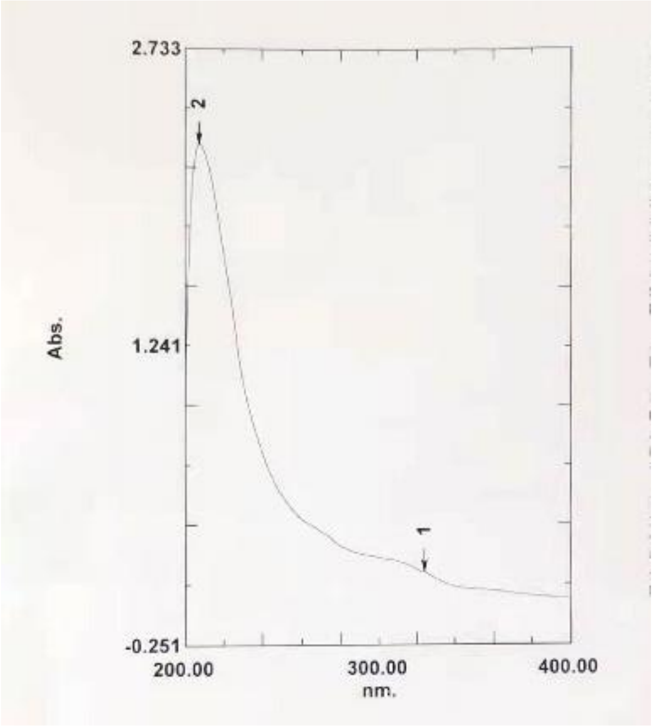

Figure S51. UV spectrum of compound **7**

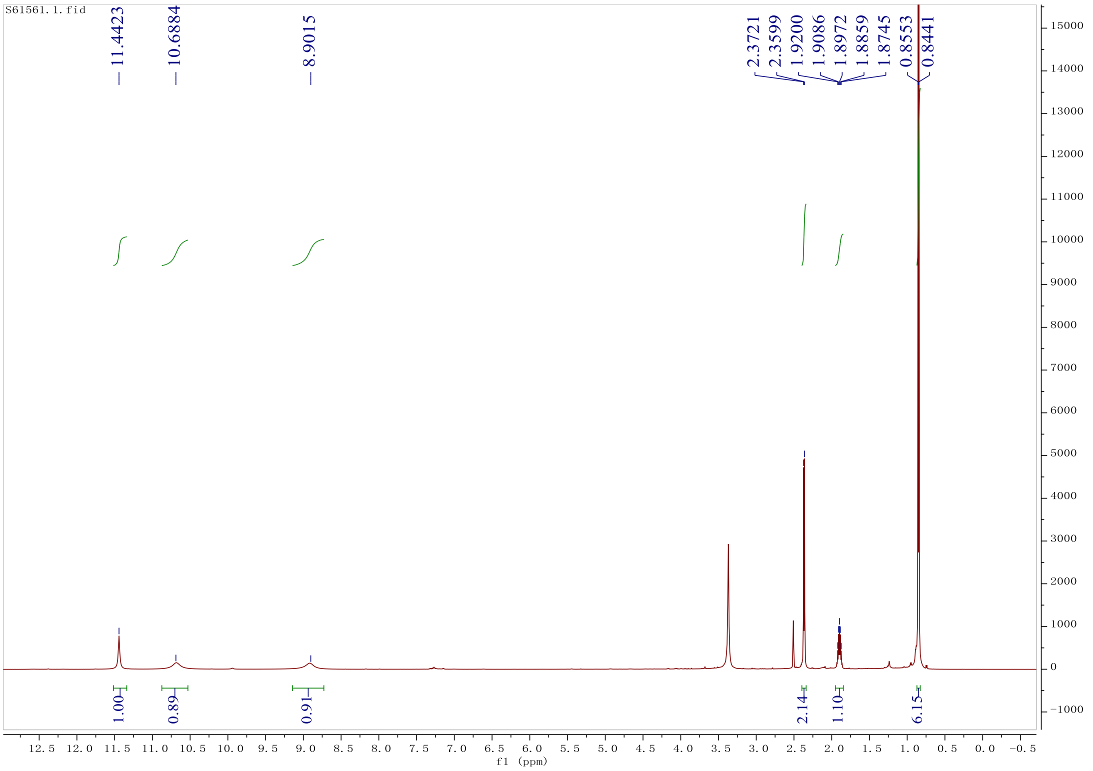

Figure S52. ^{1}H NMR spectrum of compound **8** (MDSO-d_6, 600 MHz)

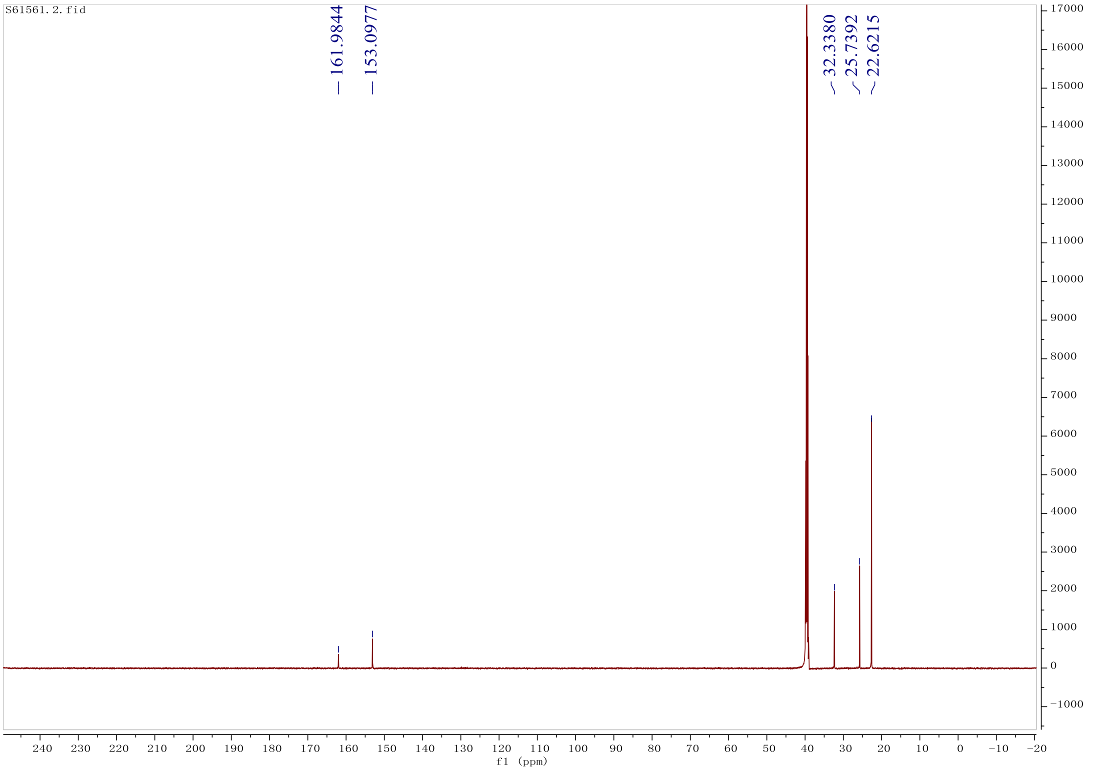

Figure S53. ^{13}C NMR spectrum of compound **8** (MDSO-d_6, 150 MHz)

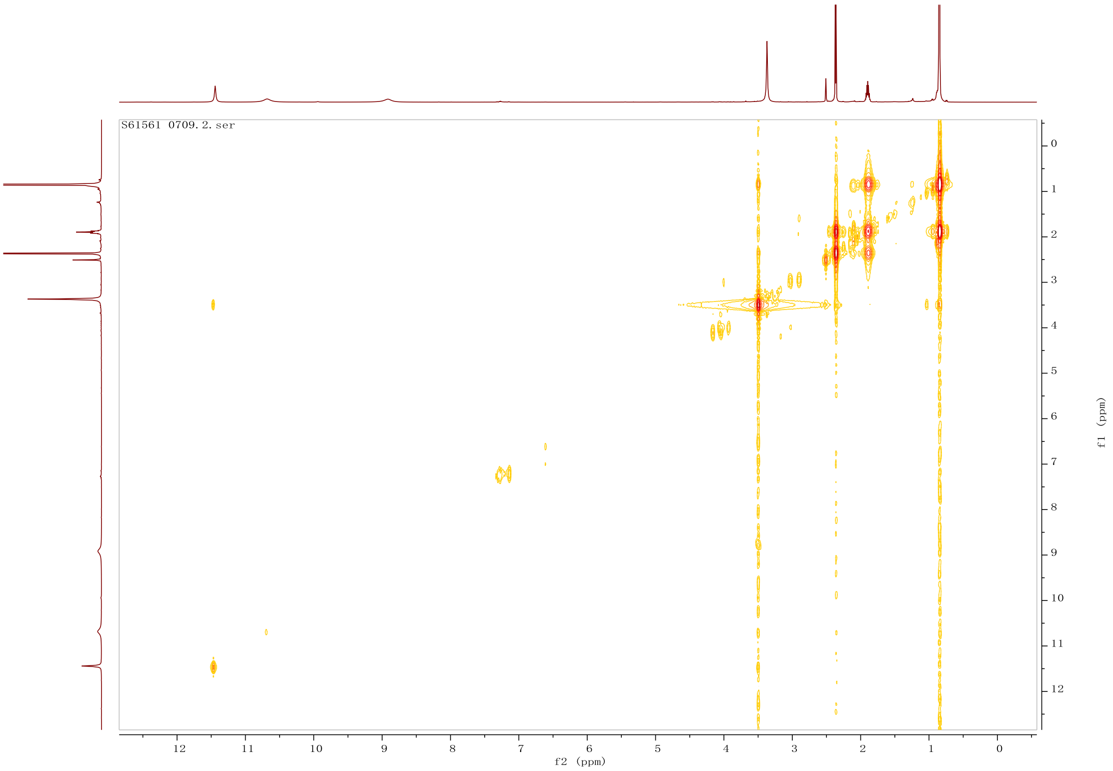

Figure S54. ^{1}H-^{1}HCOSY spectrum of compound **8** (MDSO-d_6)

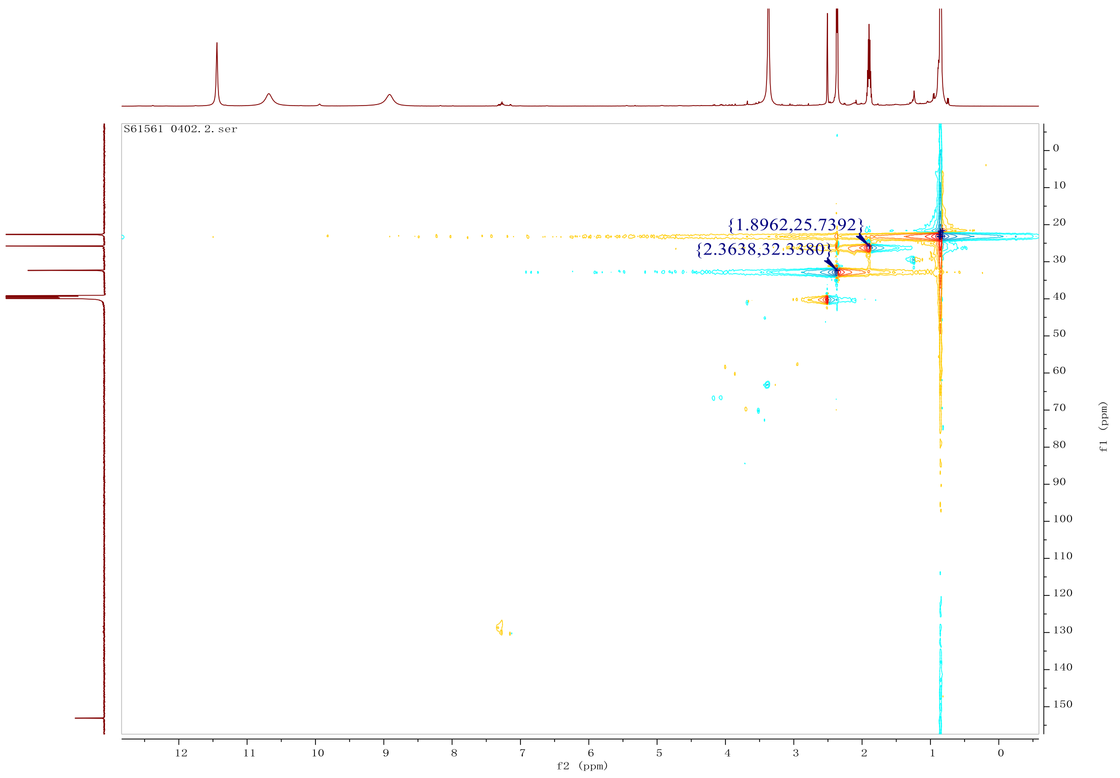

Figure S55. HSQC spectrum of compound **8** (MDSO-d_6)

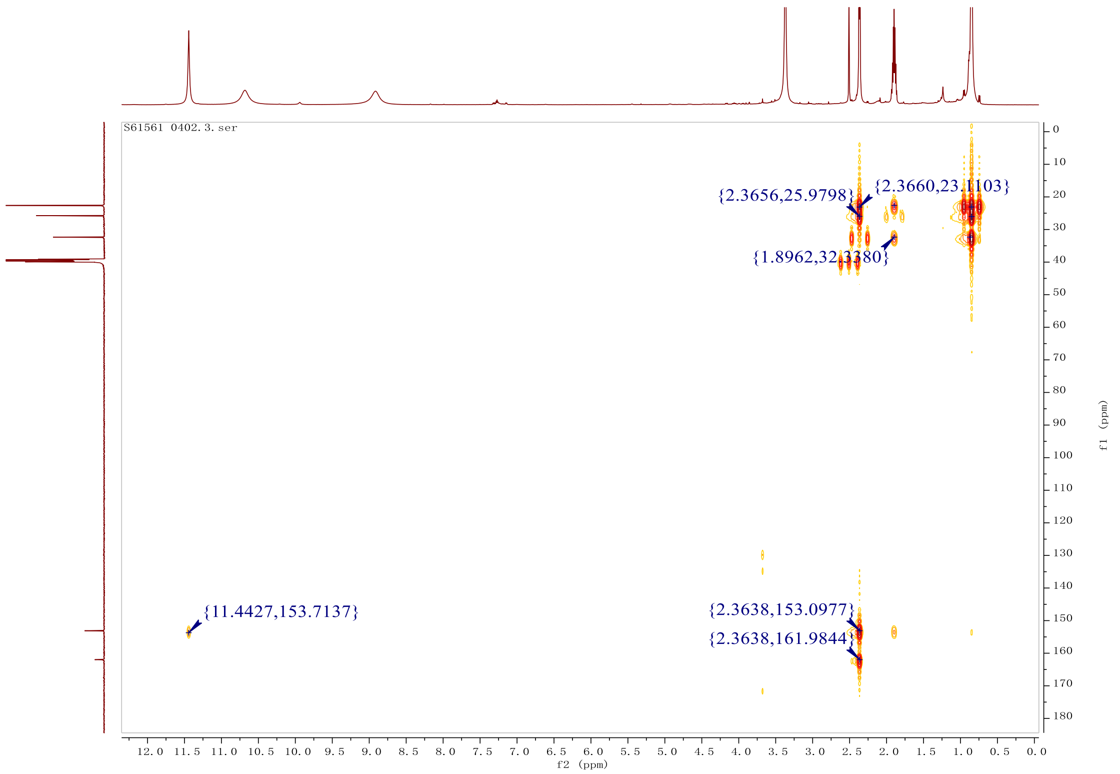

Figure S56. HMBC spectrum of compound **8** (MDSO-d_6)

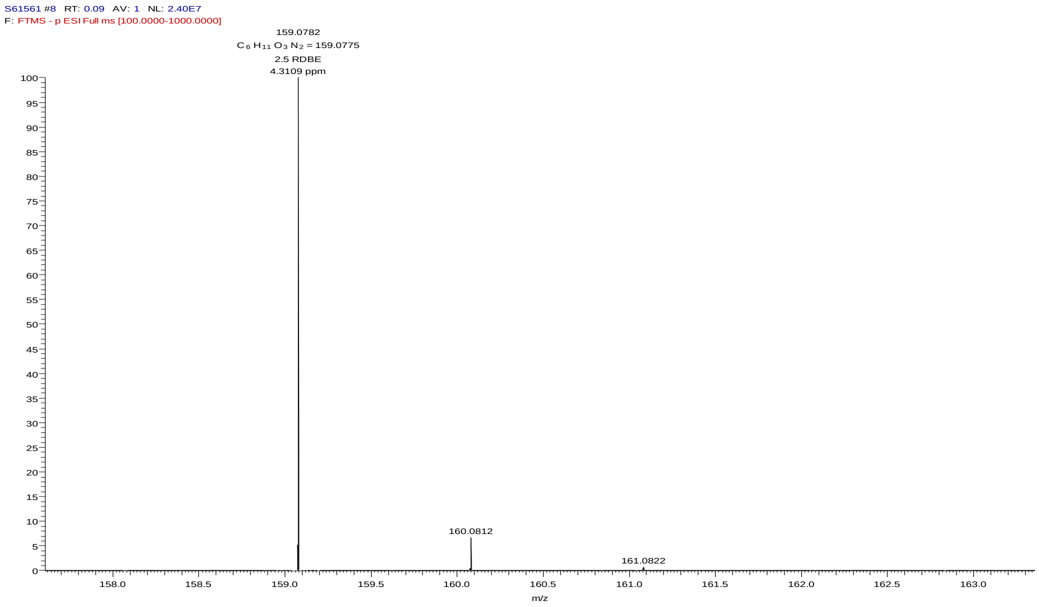

Figure S57. HRESIMS spectrum of compound **8**

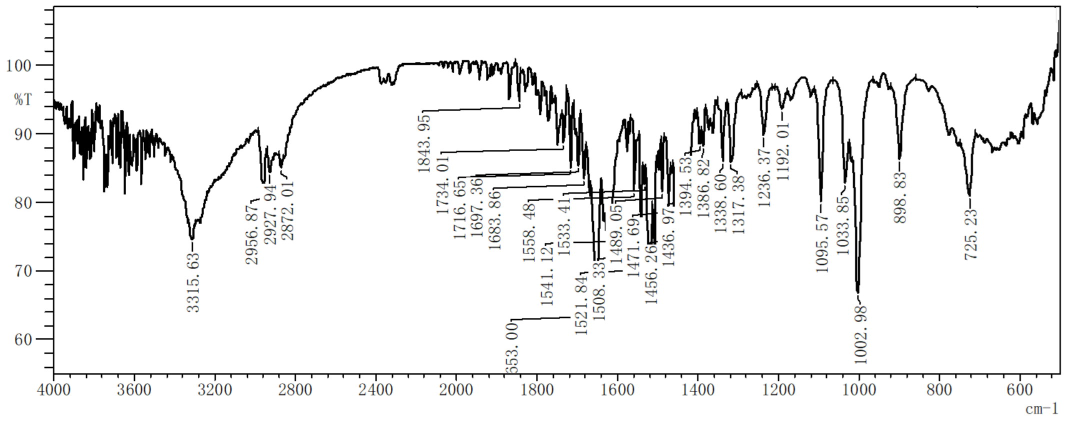

Figure S58. IR spectrum of compound **8**

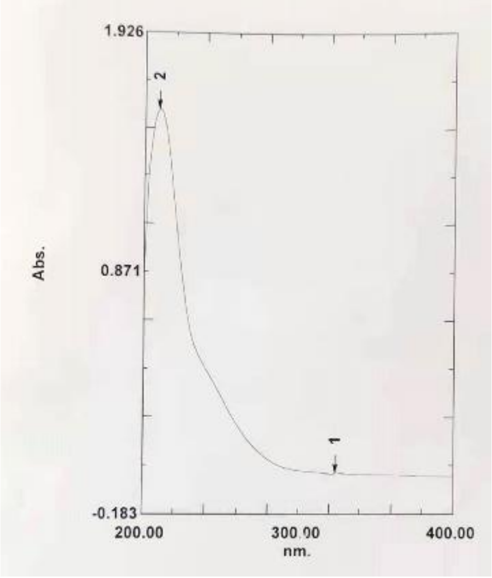

Figure S59. UV spectrum of compound **8**

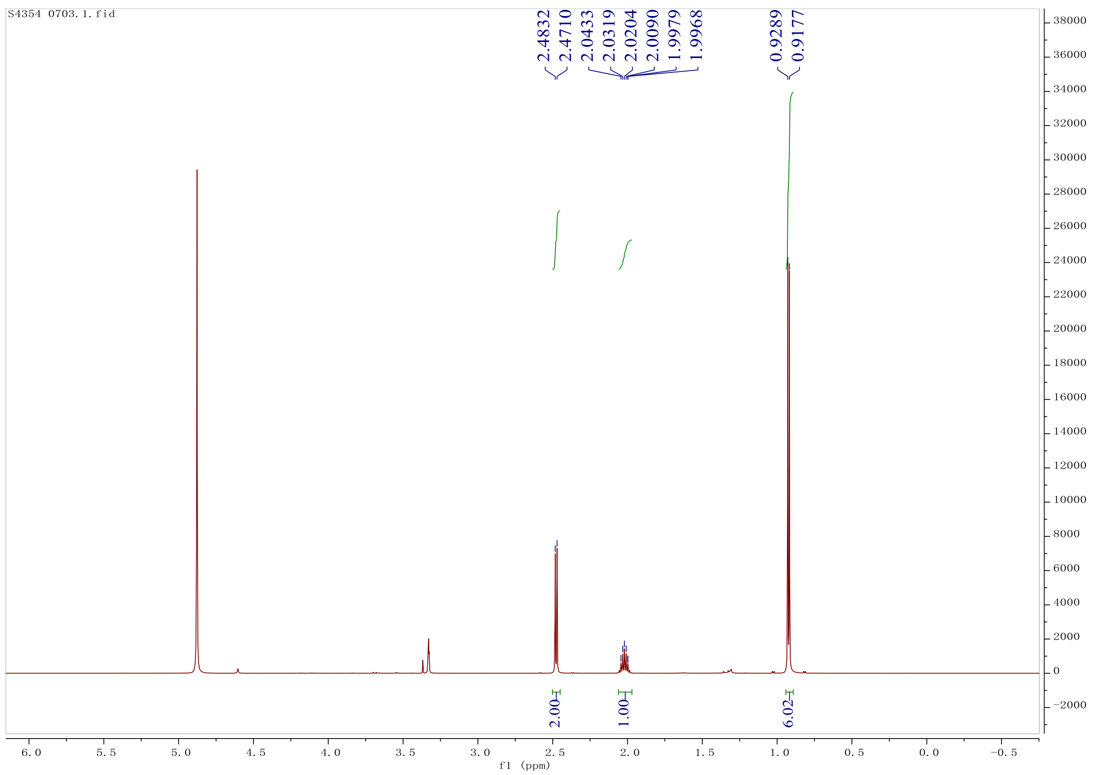

Figure S60. ^{1}H NMR spectrum of compound **9** (CD_3OD, 600 MHz)

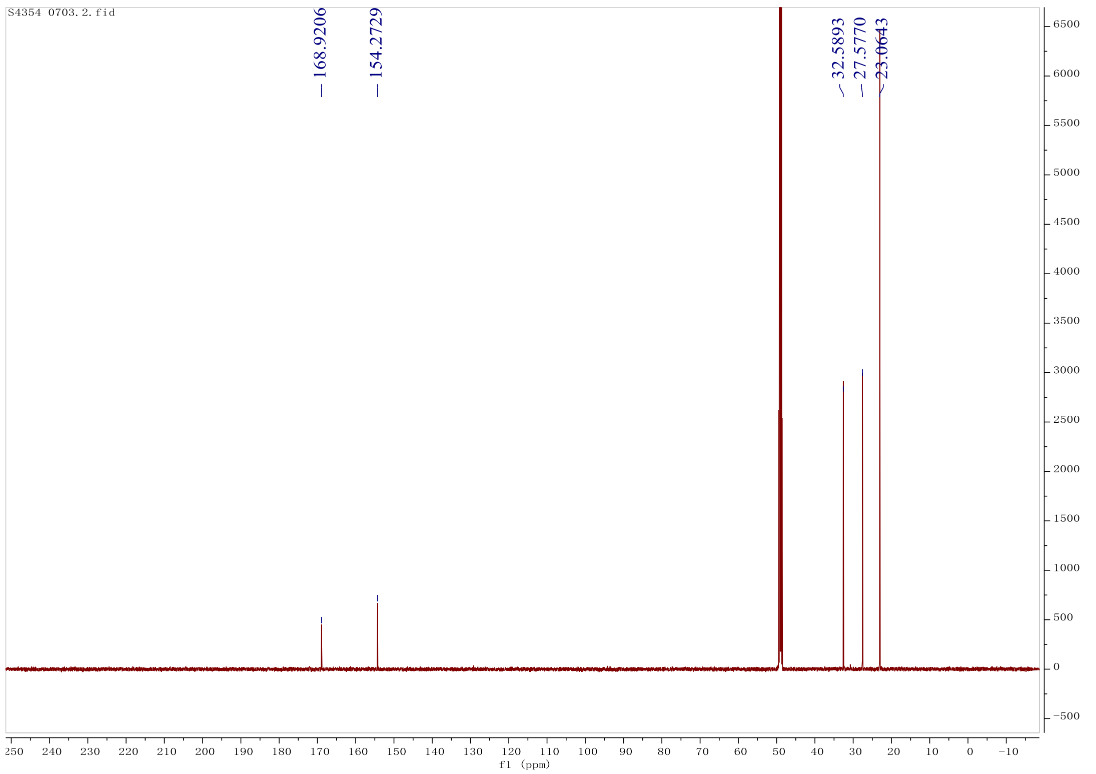

Figure S61. ^{13}C NMR spectrum of compound **9** (CD_3OD, 150 MHz)

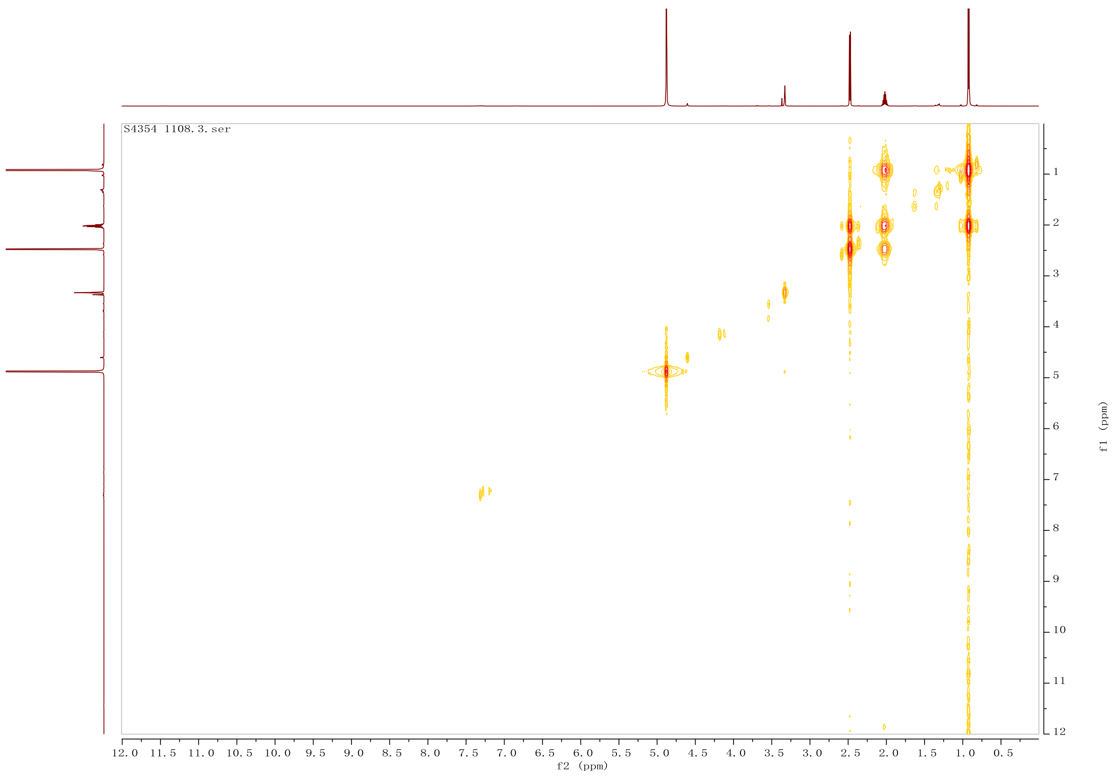

Figure S62. ^{1}H-^{1}HCOSY spectrum of compound **9** (CD_3OD)

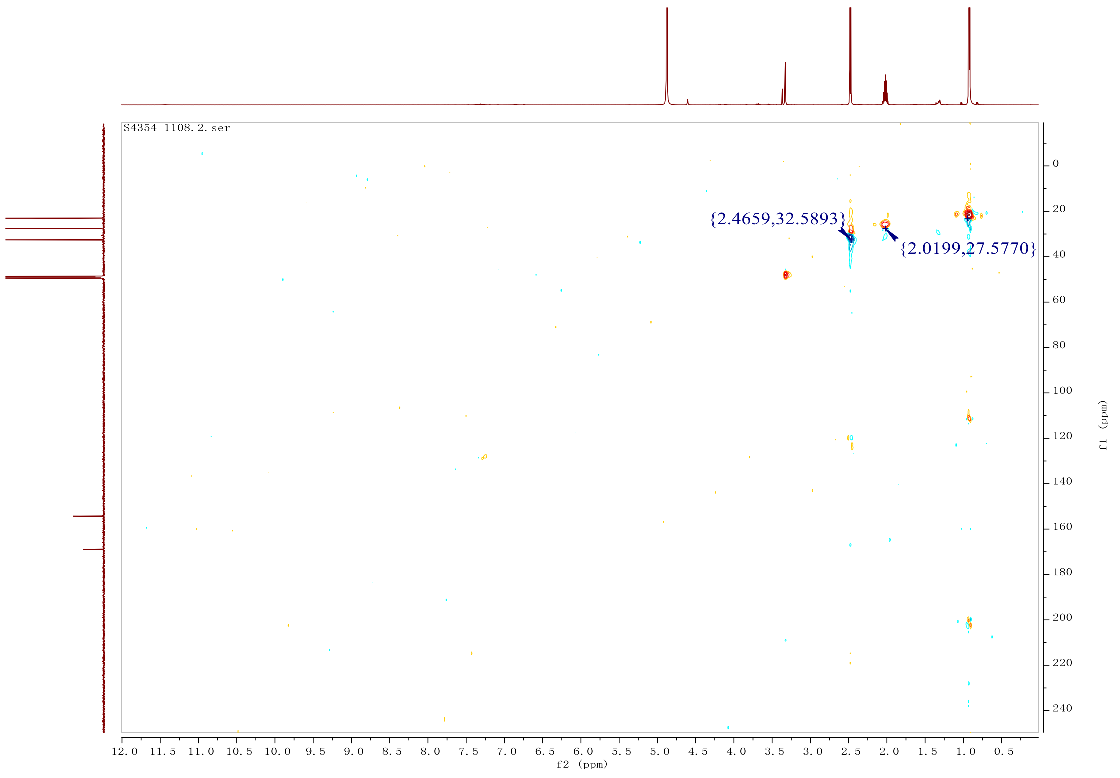

Figure S63. HSQC spectrum of compound **9** (CD_3OD)

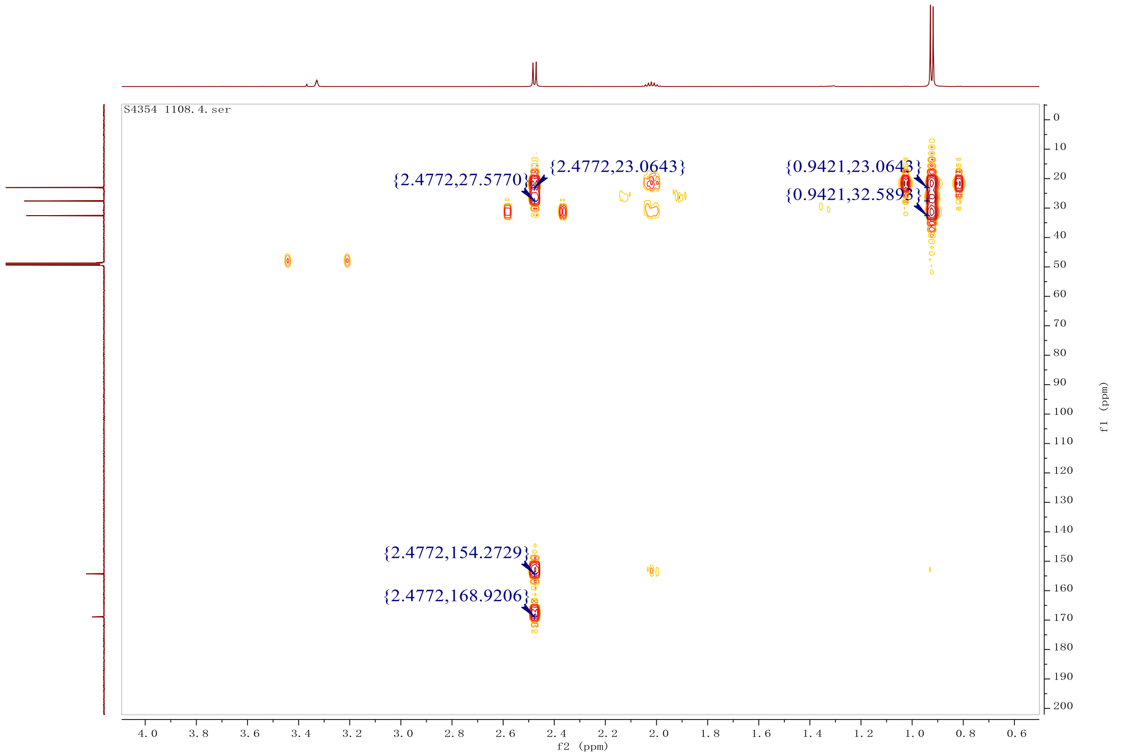

Figure S64. HMBC spectrum of compound **9** (CD_3OD)

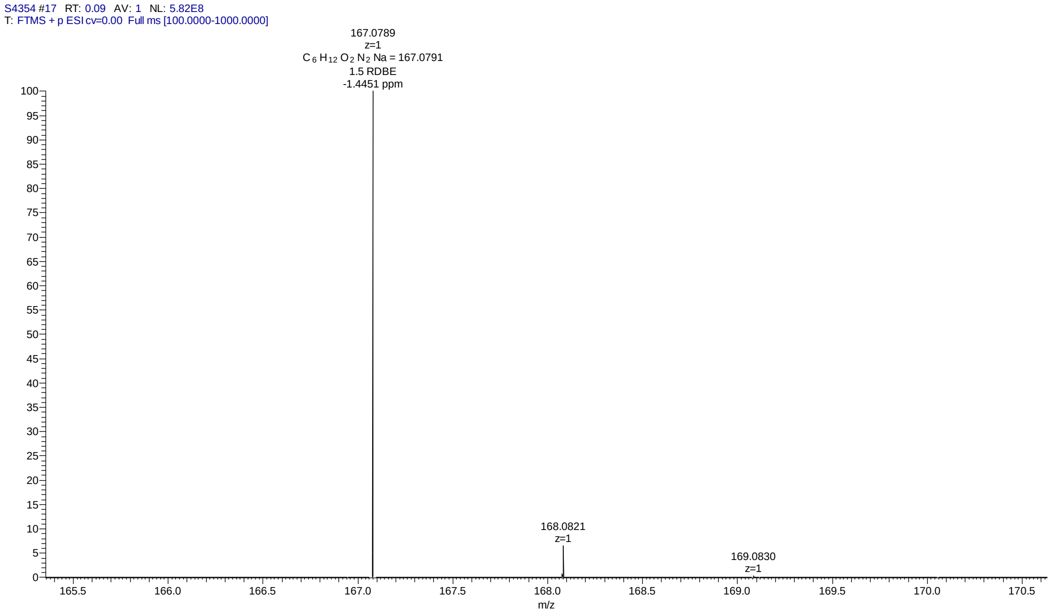

Figure S65. HRESIMS spectrum of compound **9**

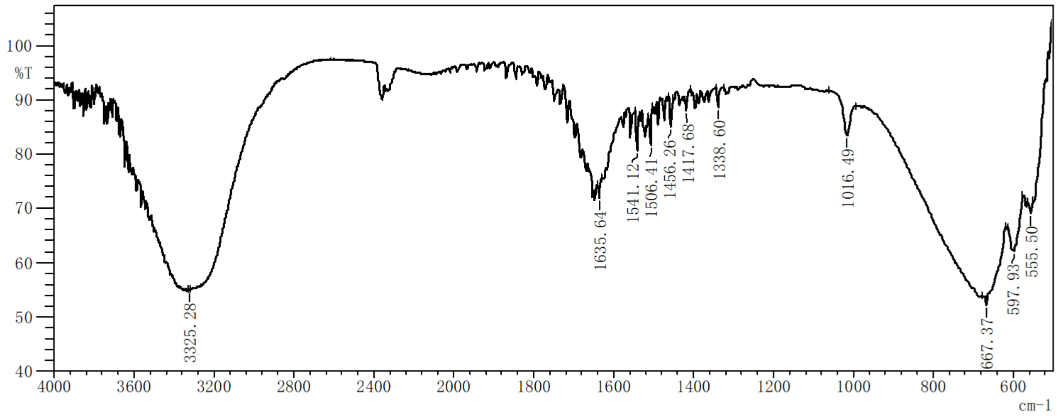

Figure S66. IR spectrum of compound **9**

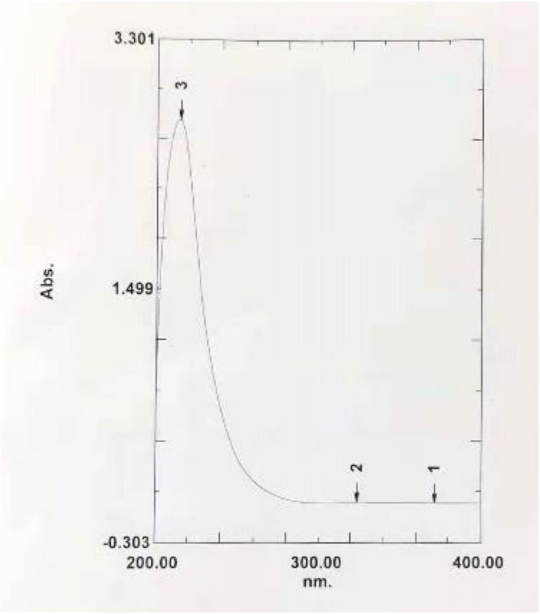

Figure S67. UV spectrum of compound **9**

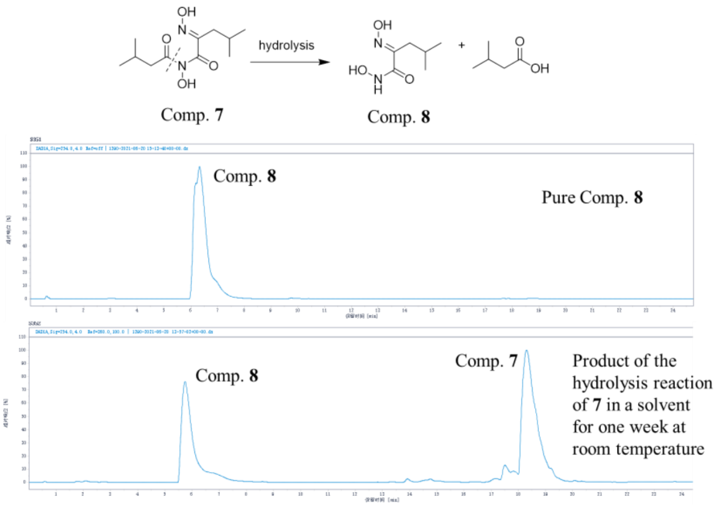

Figure S68. Hydrolysis reaction of compound **7**

Table S1. Cell Viability (%) of HepG2 treated with compounds **1-9** and lovastatin (5, 10, and 20 μM).

Compounds / Concentration(μM)	**1**	**2**	**3**	**4**	**5**	**6**	**7**	**8**	**9**	**lovastatin**
5	109.56	109.56	109.31	100.06	107.19	102.75	100.56	101.04	109.52	100.84
10	108.69	105.81	105.19	100.88	107.06	100.75	104.94	109.24	116.90	100.71
20	108.25	108.69	113.69	110.44	104.58	107.38	106.63	108.64	110.88	115.46
